# π-Hole bonding in a new co-crystal hydrate of gallic acid and pyrazine: static and dynamic charge density analysis

**DOI:** 10.1107/S2052520622001457

**Published:** 2022-03-23

**Authors:** Rumpa Pal, Christian Jelsch, Koichi Momma, Simon Grabowsky

**Affiliations:** a Faculty of Pure and Applied Sciences, University of Tsukuba, 1-1-1 Tennodai, Tsukuba, Ibaraki 305-8571, Japan; b Institute of Inorganic Chemistry and Crystallography, Department 2 – Biology/Chemistry, University of Bremen, Leobener Str. 3, 28359 Bremen, Germany; c CRM2, CNRS, Université de Lorraine, Nancy, 54000, France; d National Museum of Nature and Science, 4-1-1 Amakubo, Tsukuba, Ibaraki, Japan; e Department of Chemistry, Biochemistry and Pharmaceutical Sciences, University of Bern, Freiestrasse 3, 3012 Bern, Switzerland

**Keywords:** cocrystal hydrate, π-hole carbon bonding, static charge density, dynamic charge density, quantum crystallography

## Abstract

A new cocrystal hydrate of gallic acid with pyrazine (4GA, Py, 4H_2_O; GA_4_PyW_4_) was obtained and characterized by single crystal X-ray diffraction. In addition to structure determination, experimental charge density analysis was carried out in terms of Multipole Modelling (MP), X-ray wavefunction refinement (XWR) and maximum entropy method (MEM). As a part of XWR, the structural refinement via Hirshfeld atom refinement was carried out and resulted in O—H bond lengths close to values from neutron diffraction. A systematic comparison of molecular conformations and aromatic interactions in this new cocrystal hydrate was performed with other existing polymorphs of gallic acid. In GA_4_PyW_4_, the two symmetry-independent gallic acid molecules have a *syn* COOH orientation and form the common (COOH)_2_ dimeric synthon. The carboxyl C atom displays the characteristics of π-holes with electropositive regions above and below the molecular plane and engages in acceptor–donor interactions with oxygen atoms of acidic O—H groups and phenol groups of neighbouring gallic acid molecules. The signature of the π-hole was identified from experimental charge density analysis, both in static density maps in MP and XWR as well as dynamic density in MEM, but it cannot be pinned down to a specific atom–atom interaction. This study presents the first comparison between an XWR and a MEM experimental electron-density determination.

## Abstract 2

The π-hole carbon bonding in a new hydrate cocrystal of gallic acid with pyrazine was characterized by static and dynamic charge density analysis.

## 1. Introduction

Gallic acid (3,4,5-tri­hydroxy­benzoic acid) is a common active pharmaceutical ingredient (API) (Jyothi *et al.*, 2019 ▸). It exhibits antioxidant, antimicrobial and anticancer properties, and it is used in therapeutic applications for inflammatory allergic diseases (Choubey *et al.*, 2015 ▸). One efficient way of improving physicochemical properties such as crystallinity, melting point, solubility, dissolution, and stability of APIs without compromising the structural integrity of the API is the process of cocrystallization (Prasad *et al.*, 2015 ▸; Schultheiss & Newman, 2009 ▸). Cocrystals are composed of two or more neutral molecules with a defined stoichiometric ratio linked through various non-covalent interactions such as hydrogen bonds, van der Waals forces and aromatic stacking interactions. Pharmaceutical cocrystals are a subgroup where an API is one of the molecules of the cocrystal and it draws a considerable interest in crystal engineering and in drug discovery and pharmaceutical industries (Schultheiss & Newman, 2009 ▸).

Pharmaceutical cocrystallization often leads to hydrate formation. Because of their small size and multidirectional hydrogen bonding capacity, water molecules can easily be inserted into a crystal structure. The water environment in molecular crystals is an important source to understand the crystal packing of hydrates (Gillon *et al.*, 2003 ▸; Hickey *et al.*, 2007 ▸). Five monohydrates, three anhydrates and over 20 different solvates are already reported for the gallic acid molecule (Braun *et al.*, 2013 ▸). The crystal energy landscapes for anhydrous and monohydrate forms of gallic acid exhibit numerous thermodynamically feasible structures with a wide range of packing motifs. In the fifth blind test for Crystal Structure Prediction (CSP2010), the prediction of additional unpublished polymorphs of a hydrate was given as a challenge for the first time. The challenge was to predict the third and fourth polymorphs of gallic acid monohydrate. The target structures were published afterwards showing remarkable hydrate polymorphism (Clarke *et al.*, 2011 ▸).

Gallic acid has multiple hydrogen bonding sites. The CO moiety in the carb­oxy­lic acid has a strong hydrogen bonding acceptor property and the acidic O—H carboxy hydrogen atom has a strong hydrogen-bonding donor property. In addition, the three hydroxyl groups can act as both hydrogen-bonding donor and acceptor. The p*K_a_* value of gallic acid is 4.4. Based on p*K_a_* differences with acidic or basic coformers, gallic acid has the ability to form both salts and cocrystals (Jyothi *et al.*, 2019 ▸; Childs *et al.*, 2007 ▸). In the various polymorphs of gallic acid, the most common packing motif is the acid−acid homosynthon. The carboxyl C atom displays the characteristics of π-holes with electropositive regions above and below the molecular plane. The double π-hole characteristics of the acid dimer in a solvate of gallic acid with dioxane were recently addressed (Prohens *et al.*, 2019 ▸). In the same study, a detailed Cambridge Structural Database (CSD) analysis on aromatic carb­oxy­lic acid dimers was performed and it was demonstrated that the centrosymmetric hydrogen-bonded carb­oxy­lic dimer is well suited to form π-hole interactions (Prohens *et al.*, 2019 ▸). The analysis also showed that in 232 out of 497 investigated structures the carb­oxy­lic acid dimers participate in two symmetrically-related π-hole interactions, above and below the molecular plane. However, in the case investigated here, this symmetric motif is not present so that we investigate the characteristics of three different dimers in the crystal packing denoted dimer1, dimer2 and dimer3.

The ‘σ-hole’ concept was originally introduced to explain the apparent anomaly in halogen bonding in which an electronegative halogen (a group 17 atom) interacts attractively with a negative site (Clark *et al.*, 2007 ▸). Later, it became applicable to covalently bonded atoms in groups 16, 15 and 14 (Murray *et al.*, 2007 ▸; Politzer *et al.*, 2010 ▸) and these interactions are widely known as chalcogen, pnicogen and tetrel bonds, respectively. σ-Hole carbon bonding was investigated using theoretical and experimental charge density analysis (Mani & Arunan, 2013 ▸; Thomas *et al.*, 2014 ▸). The σ-holes are located along the extension of a covalent bond. The concept of σ-holes was extended with the description of π-holes which have analogous properties (Politzer *et al.*, 2010 ▸, 2013 ▸; Murray *et al.*, 2012 ▸). π-Holes are regions of electron density depletion that are perpendicular to portions of a molecular framework. Both positive σ-holes and π-holes can interact with negative sites, such as lone pairs, anions or π-electron systems in a highly directional manner (Pal *et al.*, 2015 ▸; Shukla *et al.*, 2018 ▸). The π-hole interaction in carbonyl compounds has been known for a long time. It was described by Bürgi and Dunitz within the famous work of the trajectory for nucleophilic attack on carbonyl groups (Bürgi, 1975 ▸). The positive electrostatic potentials above acyl carbon atoms in H_3_C—C(=O)F and H_3_C—C(=O)NH_2_ were shown to correlate with their relative tendencies to undergo hydrolysis (Sjoberg & Politzer, 1990 ▸). Other examples of π-hole containing systems are SO_2_ and SeO_2_ (Murray *et al.*, 2012 ▸; Zhang *et al.*, 2018 ▸).

Intermolecular interactions in aromatic systems are often loosely designated as π−π interactions, which is misleading. Instead, electrostatic considerations in polarized π-systems are more suitable to understand such interactions (Cockroft *et al.*, 2005 ▸; Martinez & Iverson, 2012 ▸). Also, it was shown that C⋯C contacts in crystal packings are more likely to occur for heterocyclic compounds compared to pure hydro­carbons, as stacking interactions are more favourable in the former case, due to the possibility of better electrostatic complementarity (Jelsch *et al.*, 2014 ▸).

The most suitable structural characterization technique for pharmaceutical cocrystals is X-ray diffraction. The detailed chemical information from X-ray diffraction experiments could be extracted by charge density methodologies. The chemical bonding analysis for covalent bonds and intermolecular interactions is a pillar of the emerging field of quantum crystallography which is an amalgamation of diffraction measurements and theory (Genoni *et al.*, 2018 ▸; Grabowsky *et al.*, 2017 ▸). In this study, a new pharmaceutical cocrystal hydrate of gallic acid with pyrazine was obtained and characterized by single crystal X-ray diffraction.

The p*K_a_* difference of the basic pyrazine and acidic proton in gallic acid matches well with the Δp*K_a_* rule assessing the formation of salt or cocrystal (Prasad *et al.*, 2015 ▸; Childs *et al.*, 2007 ▸). The p*K_a_* of the acidic proton in gallic acid is 4.41 and that of the N atom in pyrazine is 0.6. Hence, Δp*K*_*a*_ = p*K_a_* (base) − p*K_a_* (acid) = −3.81 is negative, which is in favour of the cocrystal formation, and not of a proton transfer from the COOH group towards the pyrazine nitro­gen.

The crystal packing involving hydrogen bonds of acid dimers and water molecules of hydration as well as aromatic stacking interactions were investigated in terms of Hirshfeld surface analysis (Spackman & Byrom, 1997 ▸; Spackman & Jayatilaka, 2009 ▸) and in terms of derived properties such as the contact enrichment ratio (Jelsch *et al.*, 2014 ▸) and energy frameworks (Turner *et al.*, 2014 ▸; Mackenzie *et al.*, 2017 ▸). Cohesive energies were calculated using periodic density functional theory (DFT) methodologies (Cutini *et al.*, 2016 ▸). In addition, a high-resolution experimental charge-density measurement was carried out. The underlying static and dynamic electron density distributions were modelled and investigated using quantum crystallographic approaches, namely, multipole (MP) model (Hansen & Coppens, 1978 ▸) and X-ray wavefunction refinement (XWR) (Grabowsky *et al.*, 2012 ▸; Woińska *et al.*, 2017 ▸) for static density and maximum entropy method (MEM) (Collins, 1982 ▸; Sakata & Sato, 1990 ▸) for dynamic density. Both the static and dynamic density descriptions unequivocally describe the signature of π-hole carbon bonding in the acid dimers of the new cocrystal GA_4_PyW_4_.[Chem scheme1] 
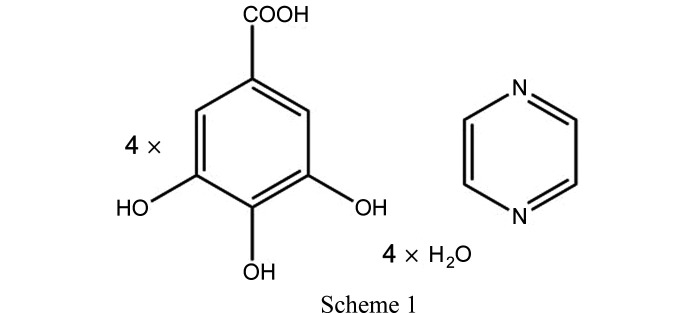


## 2. Experimental and computational details

### 2.1. Crystallization

Commercially available gallic acid (3,4,5-tri­hydroxybenzoic acid) and pyrazine were purchased from Sigma Aldrich. GA_4_PyW_4_ crystals were formed from non-dried ethanol solvent with 1:1 mixture of the two components upon slow evaporation.

### 2.2. Single crystal data collection and independent atom model refinement

**Figure 1:**
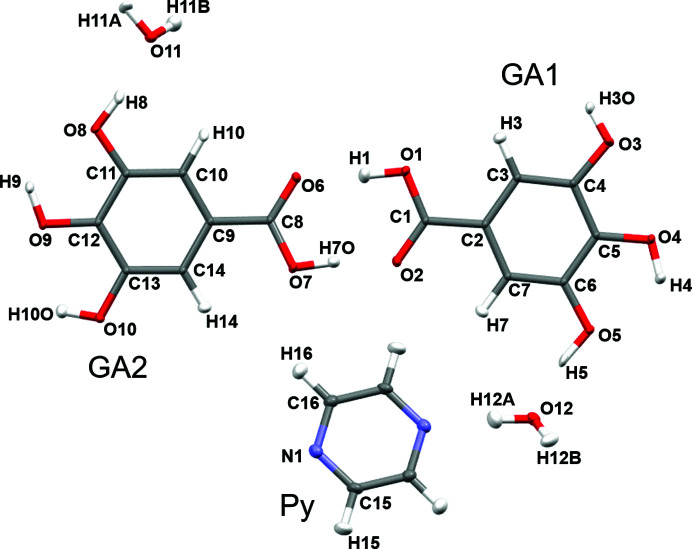
Asymmetric unit (plus half a pyrazine molecule) of the co-crystal hydrate GA_4_PyW_4_ with the atom numbering scheme (experimental multipole model geometry). This unit with a complete pyrazine molecule was considered for HAR and XCW fitting. Only the asymmetric unit atoms of pyrazine are labelled. Anisotropic displacement parameters are shown as ellipsoids at the 50% probability level.

A single crystal of size 0.13 mm × 0.15 mm × 0.25 mm was chosen using a polarizing microscope and mounted on a goniometer head using perfluorinated silicon oil. X-ray diffraction data were collected on a Bruker Venture D8 four-circle diffractometer with a Complementary Metal–Oxide–Semiconductor (CMOS) area detector Photon 100 and a microfocus source using Mo *K*α radiation (λ = 0.71073 Å) at 100 K in shutterless mode. The crystal to detector distance was fixed at 40 mm, and the scan width (Δω) was 0.5° per frame during the data collection. The data collection strategy was chosen in such a way as to yield a dataset up to *d* = 0.5 Å resolution, having both high- and low-angle frames. Unit-cell refinement, data integration and reduction, with face indexing for accurate numerical absorption correction, were carried out using the *APEX3* software (Bruker, 2015 ▸). Data merging and space group determination were performed by *XPREP* (Bruker, 2004 ▸). The crystal structure was solved by *SHELXT* (Sheldrick, 2015 ▸) and refined based on structure factor magnitudes *F* ^2^ according to the independent atom model (IAM) using *SHELXL97* (Sheldrick, 2008 ▸) included in the *WinGX* suite (Farrugia, 2012 ▸). The hydrogen atoms of COOH, phenolic groups and water molecules were located from difference Fourier maps and other aromatic hydrogen atoms were fixed stereochemically. The positions and isotropic displacement parameters of all H atoms were allowed to refine in the IAM. The crystallographic details are listed in Table 1 ▸ and the asymmetric unit of the compound is shown in Fig. 1 ▸. The asymmetric unit contains two symmetry-independent gallic acid molecules, hereafter labelled as GA1 and GA2, two water molecules and half a pyrazine molecule.

### 2.3. Multipole refinement

After the routine *SHELXL* treatment, the charge density modelling was performed according to the Hansen and Coppens multipole formalism within *MoProSuite* (Jelsch *et al.*, 2005 ▸). The least-squares minimization was carried out using all reflections. Chemical equivalence and local symmetry restraints were applied to the charge density (the weight of the restraints was *W*_r_ = 1.76/σ^2^, with σ = 0.01). The core and valence scattering factors of all atoms were derived from the Su & Coppens (1998 ▸) relativistic wavefunctions. Initially, the scale factor was refined; then positional and atomic displacement parameters were refined against all reflections. The *X*—H bond lengths were constrained to the standard values determined by neutron diffraction experiments (Allen & Bruno, 2010 ▸). The isotropic displacement parameters for H atoms were riding on their carrier atom, *U*_iso_ = 1.2*U*_eq_ (CH_2_, CH, NH) or 1.5*U*_eq_ (CH_3_, OH). For H atoms, the bond directed dipole (*dz*) component was allowed to refine. For non-hydrogen atoms, multipole populations *P*_lm_ were modelled up to the octupole level (*l* = 3).

The different charge density parameters *P*_lm_, κ′, *P*_val_ and κ were introduced in a stepwise manner in the refinement. The scale factor, positional, and anisotropic displacement parameters, *P*_lm_, *P*_val_, κ and κ′ were refined successively, until convergence was reached. Near convergence, the model was used to calculate anisotropic displacement parameters (ADPs) of H atoms using the SHADE3 server analysis (Madsen & Hoser, 2014 ▸). Estimated ADPs for H atoms were kept fixed during the subsequent multipole refinements. Keeping electroneutrality for the entire asymmetric unit, charge transfer between all the five different molecular units was allowed during the refinement. In the final model, the individual charges were: GA1: 0.02 e, GA2: 0.10 e, water_O12: −0.03 e, water_O11: 0.0 e and pyrazine (half): −0.09 e. The *VMoPro* tool was used to generate residual, deformation, and Laplacian plots and to analyse the electron lone pairs. The crystallographic refinement statistics are listed in Table 1 ▸.

### 2.4. ELMAM2 electron density model

A cheap and efficient alternative way of obtaining a multipole model is transferring multipoles from predefined databases. ELMAM2 is a database of experimentally derived multipolar atom types (Zarychta *et al.*, 2007 ▸; Domagała *et al.*, 2012 ▸). The automatic charge density transfer (multipole parameters and κ, κ′) from the ELMAM2 database was performed using the *MoPro* program. The position and displacement parameters of the atoms were kept fixed to the experimental multipolar model. The total charge of the asymmetric unit after the transfer was corrected for charge neutrality by uniformly shifting the monopole populations as implemented in the *MoPro* program.

### 2.5. Dynamic model density

The dynamic electron density was obtained by computing structure factors for a 3D grid of *N*_p_ = *N*_x_ × *N*_y_ × *N*_z_ points over the unit cell by fast Fourier transform (FFT). The series termination effect was avoided by choosing a small grid size of ∼0.05 Å (Mondal *et al.*, 2012 ▸; van Smaalen *et al.*, 2003 ▸). The dynamic density for the IAM model was computed in *VESTA* (Momma & Izumi, 2008 ▸). *MoProSuite* (Jelsch *et al.*, 2005 ▸) was used for computing the dynamic density for the ELMAM2 and MP models. The resulting density maps are denoted 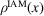 , 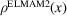 , 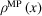 for the IAM, ELMAM2 and multipole model, respectively, and are used as prior densities in the MEM treatments (see next paragraph). Attempts to calculate dynamic model density maps after XWR resulted in rippled electron densities, so that we refrained from using those as priors for MEM studies. For smaller molecules with only one molecule in the asymmetric unit, these problems do not occur in the software *Tonto*, so we will follow up on this in a separate future study.

### 2.6. Maximum entropy method

Maximum entropy method (MEM) calculations were performed with the L-BFGS algorithm and a linear combination of generalized *F* constraints with relative weights using the *Dysnomia* package (Momma & Izumi, 2014 ▸; Momma *et al.*, 2013 ▸), considering the same grid over the unit cell as was used for the computation of the corresponding prior densities. The weighting scheme in the form of *d^x^*, where *d* is an interplanar spacing of *hkl* reflections, with *x* being 2 or 4, was tested as it is recommended for X-ray diffraction data in previous studies (De Vries *et al.*, 1994 ▸; Momma *et al.*, 2013 ▸).

Two sets of MEM densities were obtained with these two weighting schemes for each prior density. The resulting MEM densities are denoted 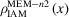 , 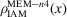 , 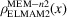 , 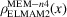 , 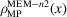 and 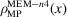 for IAM, ELMAM2 and MP prior densities. See Table 2 ▸ for details.

The weighting schemes employed in the *SHELXL97* and *MoPro* refinements were different. In this regard, *Dysnomia* uses an adjustment factor *E*, according to 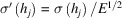 , without any conversion of 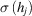 in the reflection data of various refinement models. In this study, *E* = 0.5 was used for ELMAM2 and MP prior densities whereas *E* = 1.0 for the IAM prior. *F* > 3σ(*F)* cut off criterion is used for MEM refinement.

MEM densities and dynamic model densities were visualized by three types of maps. Difference Fourier maps provide the residual density 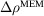 of the remaining misfit between the model and data. Dynamic deformation density is 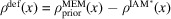 , where 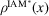 is the dynamic model density constructed from the IAM* model. This model is, on the one hand, similar to the IAM model as it does not contain any multipole and spherical charge contributions, but, on the other hand, not identical with the IAM model as it borrows atomic positions and ADPs from the respective ELMAM2 and MP priors. The third type of map is a contour map of the MEM density itself showing atomic maxima and bond critical points (BCPs).

The overall similarity between the charge density distributions was examined quantitatively by the real-space R (RSR) value (Jones *et al.*, 1991 ▸; Genoni *et al.*, 2017 ▸) defined as 

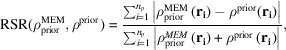

where prior stands for any of the three types of structure models, IAM, ELMAM2 or MP. RSR = 0 means complete identity.

### 2.7. X-ray wavefunction refinement

The quantum crystallographic X-ray wavefunction refinement (XWR) (Grabowsky *et al.*, 2012 ▸; Woińska *et al.*, 2017 ▸) was performed after the routine *SHELXL* treatment. In an XWR, at first a Hirshfeld atom refinement (HAR) (Jayatilaka & Dittrich, 2008 ▸; Capelli *et al.*, 2014 ▸) is carried out, followed by an X-ray constrained wavefunction (XCW) fitting (Jayatilaka & Grimwood, 2001 ▸; Grimwood & Jayatilaka, 2001 ▸). HAR is an improved structure-refinement procedure, in which tailor-made aspherical atomic scattering factors are repeatedly obtained on-the fly from an *ab initio* electron density by application of the Hirshfeld stockholder partitioning scheme (Hirshfeld, 1977 ▸). The HAR refinements were performed at RHF, BLYP and B3LYP levels with the def2-TZVP basis set with all non-H atoms as well as all hydrogen atoms being treated as anisotropic. Table 3 ▸ lists the figures of merit for these different HARs. Since the asymmetric unit contains two gallic acid, two water molecules and one half of pyrazine molecule, in HAR and XCW fitting, one full pyrazine molecule was grown along with the rest of the asymmetric unit to allow quantum-mechanical wavefunction calculations. The refinement was carried out based on structure factor magnitudes (*F*) using only reflections with *F* > 3σ(*F*). Crystal field effects were simulated by a cluster of Hirshfeld point charges for monopoles and dipoles around the central unit.

The B3LYP level of theory resulted in O—H distances closer to the standard neutron values compared to BLYP. Hence, further XCW fitting were carried out based on the fixed B3LYP geometry, but using a HF wavefunction ansatz without explicit consideration of cluster charges and dipoles. It was shown to be beneficial to use the HF approach in the XCW fitting to obtain unbiased electron correlation from the experiment (Genoni *et al.*, 2017 ▸). In the XCW fitting procedure, the external manually adjusted multiplier λ was varied from 0.0 to 2.0 with an interval of 0.05. The SCF cycles converged until λ_max_ = 0.7 and the derived wavefunction and electron density at this λ_max_ = 0.7 step were considered for subsequent charge density analysis. The full XWR was carried out with the software *Tonto* (Jayatilaka & Grimwood, 2003 ▸). The input files for XWR were prepared using the lamaGOET interface (Malaspina *et al.*, 2021 ▸). The χ^2^ agreement statistics is shown in Fig. S1 and the values of χ^2^, λ and electronic energies are listed in Table S1.

### 2.8. Topological properties of the electron density

A topological analysis according to the Quantum Theory of Atoms in Molecules (QTAIM) (Bader, 1990 ▸) of each of the MEM densities, each dynamic model density and the XWR density were performed in *MoProViewer* inside the *MoProSuite* package. For the static multipolar density, the *VMoPro* module was used inside *MoProSuite*. The density and Laplacian values of the bond critical points of covalent bonds and intermolecular interactions were obtained from 3D grids. The atomic Bader charges and volumes were also integrated.

### 2.9. Computational details

The geometry optimizations *in vacuo* for each of the two symmetry-independent gallic acid molecules in the asymmetric unit, GA1 and GA2, individually, were carried out with *Gaussian16* (Frisch *et al.*, 2016 ▸) at B3LYP-D3/6-311++G(2d,2p) level (Grimme, 2006 ▸; Grimme *et al.*, 2010 ▸; Hehre *et al.*, 1986 ▸). The optimizations converged with no imaginary frequencies. Single point calculations were also performed on the optimized geometries at B3LYP-D3/def2-TZVP level, *i.e.* with the identical basis set that was used in XWR. The QTAIM topological analysis (Bader, 1990 ▸) was performed for both GA1 and GA2 using the *AIMALL* package (Keith, 2013 ▸).

The natural bond orbital (NBO) analysis (Reed *et al.*, 1986 ▸, 1988 ▸) of the two dimers [dimer1// and dimer3] involving π-hole interactions (as described later in Fig. 5) was performed by single point calculations with experimental multipole model geometry at B3LYP-D3/def2-TZVP level using *NBO6.0* package (Glendening *et al.*, 2013 ▸) interfaced to *Gaussian16*. The *ChemCraft* visualization software (http://www.chemcraftprog.com) was utilized for plotting the natural bond orbitals between interacting atoms.

Cohesive energies (Cutini *et al.*, 2016 ▸) were calculated at B3LYP-D2/pob-TZVP_2012 level with the Grimme dispersion correction as implemented in the *CRYSTAL14* package (Dovesi *et al.*, 2014 ▸), which includes 3D periodicity. The Δ*E*_cohesive_ term corresponds to the sum of two contributions:

Δ*E*_cohesive_ = Δ*E*_cond_ + Δ*E*_conf_ (cond = condensation, conf = configuration).

Δ*E*_cond_ refers to the condensation of molecules keeping the same conformation in the crystal form and *in vacuo*. The second term, Δ*E*_conf_, accounts for the energy difference arising from the conformational change between crystal phase and isolated state. For periodic calculations, the basis set superposition error (BSSE) needs to be corrected. It affects the Δ*E*_cond_ term.

So, overall, Δ*E*_cohesive_ = Δ*E*_cond_ + Δ*E*_conf_ + BSSE.

These three contributions are calculated in the following way:

Δ*E*_cond_ = *E*(bulk)/*Z* –*E*(mol, crystal), where *Z* is the number of molecules in the unit cell,

Δ*E*_conf_ = *E*(mol, crystal) – *E*(mol, gas phase),

BSSE = *E*(mol, crystal) – *E*(mol, ghosts);

where *E*(mol, crystal) is the total energy of the molecule in the crystal geometry, *E*(mol, gas phase) is the total energy of the molecule in the optimized geometry *in vacuo* and *E*(mol, ghosts) is the total energy of the molecule in the crystal geometry with augmenting the basis set with the ghost functions of surrounding atoms, used for BSSE correction.

Δ*E*_cohesive_ was calculated for GA1 and GA2 separately. The single point energy of the unit cell, *E*(bulk), was calculated at a fully periodic level on experimental geometries with *X*—H (*X* = C, O, N) distances elongated to standard values from neutron diffraction. For *E*(mol, crystal), the energies of GA1 and GA2 with the identical geometry as in the crystal structure were extracted. The calculations were performed at B3LYP-D3/pob-TZVP-rev2 level. As a representative of *E*(mol, gas phase), the optimized geometries for GA1 and GA2 at B3LYP-D3/6-311++g(2d,2p) level obtained from *Gaussian16* were considered and the single point energies at B3LYP-D3/ pob-TZVP-rev2 level were recalculated in *CRYSTAL17*. For this multicomponent system, *Z* value was considered as 4.882 which is a representative ratio of *E*(bulk)/*E*(mol, crystal) for GA1 and GA2.

Hirshfeld surfaces, fingerprint plots (Spackman & Jayatilaka, 2009 ▸) and model energies (Turner *et al.*, 2014 ▸) were obtained from *Crystal Explorer* (Turner *et al.*, 2017 ▸). The contact enrichment ratios (Jelsch *et al.*, 2014 ▸) were obtained using the Hirshfeld surface module within *MoProViewer* software (Guillot *et al.*, 2014 ▸). The Hirshfeld surface was generated using two asymmetric units to avoid having to handle half a pyrazine molecule. Two GA1, GA2, WAT1, WAT2 and one pyrazine molecules not in contact with each other were selected in the crystal packing in order to generate an integral Hirshfeld surface around each entity.

## 3. Results and discussion

### 3.1. Molecular conformation

**Figure 2:**
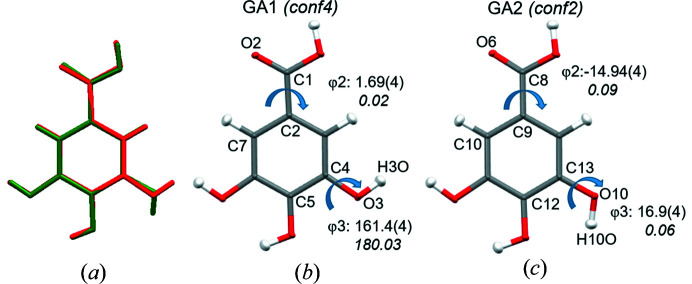
(*a*) Overlay of the two symmetry-independent molecules, GA1 and GA2 (RMSD: 0.1481 Å) after experimental multipole model, (*b*) GA1 with conformation *conf4* and (*c*) GA2 with conformation *conf2*. The following torsion angles (in °) are shown by a blue arrow: in GA1, φ_2_(C7—C2—C1—O2) and φ_3_(H3O—O3—C4—C5); in GA2, φ_2_(C10—C9—C8—O6) and φ_3_(H10O—O10—C13—C12). Values in the first line correspond to the crystal geometry (multipole model) and values in the second line correspond to the *Gaussian16* optimized structure.

An earlier report (Braun *et al.*, 2013 ▸) on an exhaustive potential energy surface (PES) scan of the flexible gallic acid molecule based on the rotation of the hydroxyl (C—C—O—H) and the carboxyl acid group (C—C—C—O) revealed four planar conformational minima. Two of them, labelled as *conf2* and *conf4* in Braun *et al.* (2013 ▸), were related by the difference in torsion angle of the *meta* OH group. In our GA_4_PyW_4_ cocrystal, the two symmetry-independent gallic acid molecules, hereafter labelled as GA1 and GA2, resemble *conf4* and *conf2*, respectively. GA_4_PyW_4_ is the only cocrystal hydrate reported so far with both conformations *conf4* and *conf2* in one crystal structure. The torsion angle of the *meta* OH group is φ_3_ = 161.4 (4)° and 16.9 (4)° in GA1 and GA2, respectively, as per experimental multipole model (Table 4 ▸). An overlay diagram of GA1 and GA2 is shown in Fig. 2 ▸. Also, the COOH group is more coplanar with the phenyl ring in GA1, compared to GA2, with φ_2_ = 1.69 (4)° and −14.95 (4)°, respectively, as per experimental multipole model (Fig. 2 ▸, Table 4 ▸).

After optimization of GA1 and GA2 separately *in vacuo*, two different optimized geometries, both minima on the PES, were found, corresponding to earlier calculations of *conf2* and *conf4*. Both the COOH group and the *m*-hydroxyl group became perfectly coplanar with respect to the phenyl ring compared to the geometries in the crystal state. In case of GA1, this resulted in a deviation of ∼18.6° for the torsion angle of the *m*-hydroxyl group (C5—C4—O3—H3O) from the crystal geometry and only ∼1.7° for the torsion angle (C7—C2—C1—O2) associated with the COOH group coplanarity. In case of GA2, the deviations were ∼16.9° and ∼14.9°, respectively, for the above two torsion angles upon geometry optimization.

The QTAIM analysis does not show any intramolecular hydrogen-bond formation. Molecular graphs with electron density and Laplacian values at bond critical points and Bader atomic charges for GA1 and GA2 are shown in Fig. S2. In the gas phase optimized geometry, GA2 is more stable by −14.8 kJ mol^−1^ compared to GA1. The cohesive energy calculations indicate that in the crystal structure, GA1 gets more stability by −31.4 kJ mol^−1^ (Table 5 ▸). The reversal of stability in the crystal state is reflected by the destabilization caused by the deviation of the COOH group from planarity with respect to the phenyl ring by |φ_2_| = 14.95 (4)° in GA2 compared to 1.69 (4)° in GA1 (Table 4 ▸). Also, the three phenolic OH groups in GA1 are involved in six hydrogen bonds with neighbouring molecules whereas those in GA2 form a total of five hydrogen bonds which are adding extra stability to GA1, as shown in Fig. S3 and Table S2.

### 3.2. Spread of O—H bond lengths from HAR

**Figure 3:**
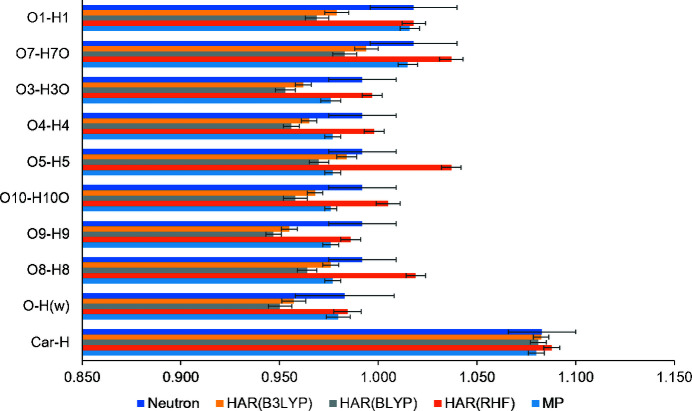
Comparison of *X*—H bond lengths (in Å) obtained from various HAR refinements and multipole models. The acidic and phenolic O—H bonds have individual entries, whereas O—H(w) is an average of four bonds and similarly Car-H is an average of all the aromatic C—H bonds. The ‘Neutron’ entry refers to averaged values from Allen & Bruno (2010 ▸). The ‘MP’ entry refers to the refined distances, but it has to be recalled that the *X*—H bond lengths were restrained to the standard neutron values.

The acidic and phenolic O—H bond lengths obtained from various HAR refinements and multipole models are compared in Fig. 3 ▸. In the multipole model, all C—H and O—H bond lengths are restrained to those from standard neutron diffraction distances (Allen & Bruno, 2010 ▸), so their interpretation is meaningless. In the HAR models, the O—H and C—H bond distances are freely refined. For the C—H bonds, the values agree between all four models. For the polar O—H bonds which are involved in intermolecular interactions, the values from the DFT models are shorter than the neutron reference values, whereby those obtained at the higher B3LYP level are always a bit longer than those from the lower BLYP level, and closer to the standard neutron diffraction distances. The O—H bond distances from HAR using Hartree–Fock are always longer than the neutron references, in two cases even significantly too long (O5—H5 and O8—H8). Overall, this indicates that the inclusion of electron correlation effects in the DFT functionals is important, and mostly beneficial for the accurate localization of hydrogen atoms. In HF, ionic resonance forms are normally overestimated which explains why they are longer than the neutron references. However, we cannot explain why on average the HF results are closer to the neutron reference values than the DFT results. Other bond lengths, bond angles and torsion angles for GA1 and GA2 are listed in Tables S3 and S4, respectively.

### 3.3. Crystal packing

**Figure 4:**
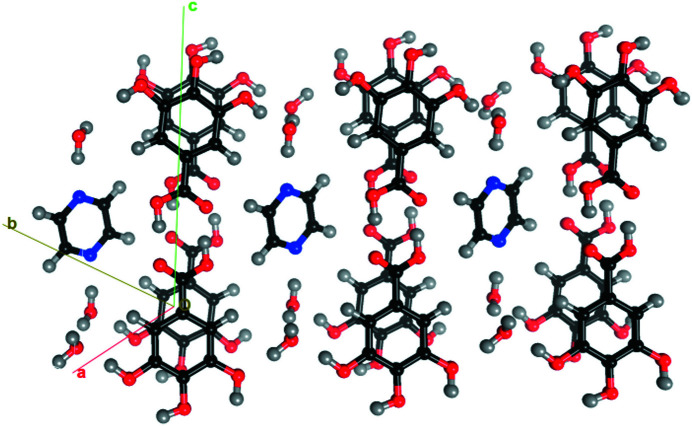
Crystallographic autostereogram of the GA_4_PyW_4_ packing, generated with *MoProViewer* (Guillot *et al.*, 2014 ▸). Two independent layers of gallic acid molecules, forming parallel stacking, are shown along the **a** − **b** axes. Horizontal translations correspond to the **a**+**b** vector.

The crystal structure is stabilized by various strong hydrogen bonds and aromatic stacking interactions. The projection along the **b** direction indicates that aromatic stacking layers of gallic acid molecules are separated from each other through a channel of alternating water and pyrazine molecules connected by strong O—H⋯O and O—H⋯N hydrogen bonds among each other (Fig. 4 ▸).

The two symmetry-independent gallic acid molecules GA1 and GA2 have both *syn* COOH conformation (Pal *et al.*, 2018 ▸) and form an acid homo-dimer (Fig. 1 ▸). The hydrogen-bond geometries for the acid dimeric motifs as obtained from three types of HAR refinements and the multipole model are listed in Table S5. Since determination of H atom positions is sensitive compared to the rest of the atoms, the O—H and H⋯O distances turned out to be slightly different in various models, but O⋯O distances and ∠O—H⋯O angles remained more similar. The charge density analysis of this dimeric motif is discussed in later sections in detail.

Hirshfeld surfaces and fingerprint plots (Spackman & McKinnon, 2002 ▸) were generated using *Crystal Explorer* for all the symmetry-independent molecules in the asymmetric unit. Relative contributions to the Hirshfeld surface area for various close intermolecular contacts (Fig. S4) reveal that, for the two symmetry-independent molecules GA1 and GA2, the dominant contacts are O⋯H (48.1% and 45.0%) and H⋯H (23.2% and 25.9%), respectively. There is a slight difference in the relative contributions of O⋯C, C⋯C and C⋯H contacts for the two gallic acid moieties. The energy frameworks are shown in Fig. S5.

The different contact types and their enrichment in the crystal packing were also further analyzed with *MoProViewer* (Guillot *et al.*, 2014 ▸). The enrichment ratio *E*_*XY*_ for a pair of elements (*X*,*Y*) is defined as the ratio between the proportion of actual contacts *C*_*xy*_ in the crystal and the theoretical proportion *R*_*xy*_ of equi-distributed random contacts (Jelsch *et al.*, 2014 ▸). An enrichment ratio larger than unity reveals that a contact type is over-represented in the crystal, while pairs which tend to avoid contacts with each other should yield an *E* value lower than unity. In order to obtain integral Hirshfeld surfaces around all entities, a large cluster of molecules was generated. Molecules not in contact with each other in the crystal packing were selected. Since only half of a pyrazine molecule is present in the asymmetric unit, the statistical analysis of the contacts was performed on two asymmetric units, to consider the full pyrazine molecule in the calculation.

The nature of the intermolecular contacts and their enrichments in the crystal structure are shown in Table 6 ▸. The major contact type is O—H⋯O and the strong hydrogen bonds, O—H⋯O, O—H⋯N as well as C⋯C contacts are enriched. These facts support that the crystal structure is primarily stabilized by strong hydrogen bonds and extensive aromatic stacking. 39.7% of the surface is hydro­phobic, made of non-polar atoms C and Hc (H atoms bonded to carbon) and over-represented by *E* = 1.36. These dispersion interactions participate in the crystal packing stabilization. Hc⋯Hc contacts exist between pyrazine and the two gallic acid molecules. C⋯Hc is the only under-represented hydro­phobic contact, as there are no C-H⋯π weak hydrogen bonds and all the gallic acid and pyrazine molecules have aromatic planes which have similar orientations. The two gallic acid molecules are nearly parallel. In a computation of the Hirshfeld surface around each of two independent gallic acid moieties, the proportion of contact types *C*_*xy*_ were found to be 94.1% correlated on the two moieties.

Contacts between hydro­philic atoms are also enriched but to a smaller extent (*E* = 1.20). Whereas cross contacts between hydro­philic and hydro­phobic atoms (HPL*HPB) are disfavored (*E* = 0.73). O⋯Hc weak hydrogen bonds are the only cross contacts between hydro­philic and hydro­phobic atoms which are enriched (*E* = 1.08) in the crystal packing.

#### 3.3.1. Aromatic stacking interactions

**Figure 5:**
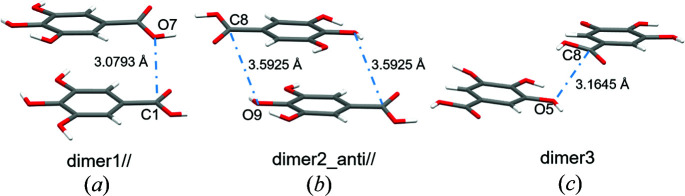
The three dimers investigated in this paper: (*a*) parallel arrangement of GA1 (below) and GA2 (above) in subsequent layers (aromatic stacking plus π-hole interaction), (*b*) antiparallel arrangement of two GA2 molecules in subsequent layers (aromatic stacking, negligible π-hole interaction), (*c*) a third arrangement of two molecules, GA1 (below) and GA2 (above) (no aromatic stacking but π-hole interaction).

Two types of aromatic stacking motifs involving gallic acid are observed in GA_4_PyW_4_; (i) parallel arrangement of GA1 and GA2 in dimer1// and (ii) antiparallel arrangement of GA2 molecules in subsequent layers in dimer2_anti//, as shown in Fig. 5 ▸. There is another dimeric motif not representing aromatic stacking, but involving a π-hole interaction, which will be discussed later, called dimer3 in Fig. 5 ▸. To understand the relative contributions of electrostatic and dispersion terms, pairwise intermolecular interaction energies were calculated with *Tonto* inside *Crystal Explorer* at the default benchmarked theoretical level B3LYP/6-31g(d,p) (Turner *et al.*, 2014 ▸). A cluster of molecules within a radius of 3.8 Å surrounding one central GA1 gallic acid molecule (and GA2, respectively) was constructed. All the dimers formed within this cluster and involving the central molecule were considered. The total interaction energy per dimer is the scaled sum of electrostatic, dispersion, polarization and exchange–repulsion terms.

It is observed that in general for both parallel and antiparallel stacking, the most contributing term towards stabilization is the dispersion contribution. Also, it is interesting to note that electrostatic interactions are stabilizing only the antiparallel stacking. To compare this trend among other reported polymorphs of gallic acid, the following four modifications were chosen: an anhydrous form, labelled as GA-I hereafter (AH-I; Braun *et al.*, 2013 ▸) and three monohydrated forms [MH-I (Okabe *et al.*, 2001 ▸; Billes *et al.*, 2007 ▸), MH-II (Demirtaş *et al.*, 2011 ▸; Clarke *et al.*, 2011 ▸) and MH-V (Braun *et al.*, 2013 ▸)], labelled as GAW-I, GAW-II and GAW-V respectively. The GA molecule adopts two conformations: *conf2* in GA-I and GAW-I, and *conf4* in GAW-II and GAW-V. It is noteworthy to mention that in *conf4*, since two hydroxyl oxygen atoms face each other, this conformer is not found in anhydrous polymorphs but is only observed in hydrated polymorphs.

**Figure 6:**
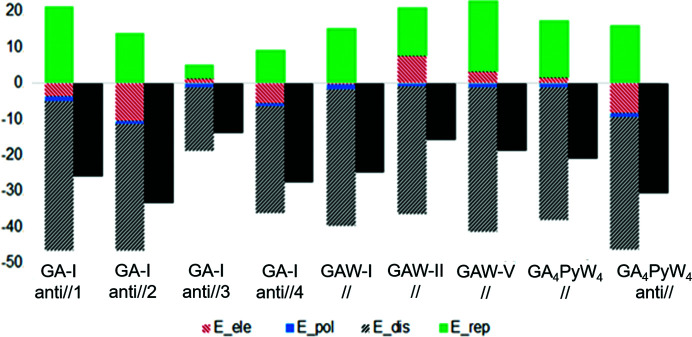
Total interaction energy (black) and its components, electrostatic, polarization, dispersion and repulsive contributions, between the different dimers interacting through aromatic stacking in the following crystal forms: GA-I (*Z*′ = 2), GAW-I, GAW-II, GAW-V and our cocrystal hydrate structure GA_4_PyW_4_. The energies (kJ mol^−1^) are scaled with respective default benchmarked scale factors for B3LYP/6-31g(d,p) level of theory (Turner *et al.*, 2014 ▸).

The individual energy components along with the total energy for various dimers engaged in parallel and antiparallel aromatic stacking are presented in Fig. 6 ▸. In GA-I, there are two symmetry-independent gallic acid molecules with conformation *conf2* in the asymmetric unit. They form four types of antiparallel aromatic stacking with neighbouring molecules, labelled as GA-I (anti//1, 2, 3 and 4) in Fig. 6 ▸. The monohydrated forms, GAW-I, GAW-II and GAW-V, have one gallic acid molecule in the asymmetry unit and they form parallel stacking interactions. The two modes, parallel and antiparallel stacking, occur together in the same crystal structure only in the GA_4_PyW_4_ cocrystal hydrate presented in this study, and they are labelled as GA_4_PyW_4_ (//) and GA_4_PyW_4_ (anti//) in Fig. 6 ▸. The relative contributions for all aromatic stackings reveal that for the antiparallel arrangement (with *conf2* conformation) of GA molecules, the electrostatic component is in general attractive (except for GA-I, anti//3). Conversely, two parallel GA stackings have unfavourable electrostatic energy while the two others have close to zero energy.

**Figure 7:**
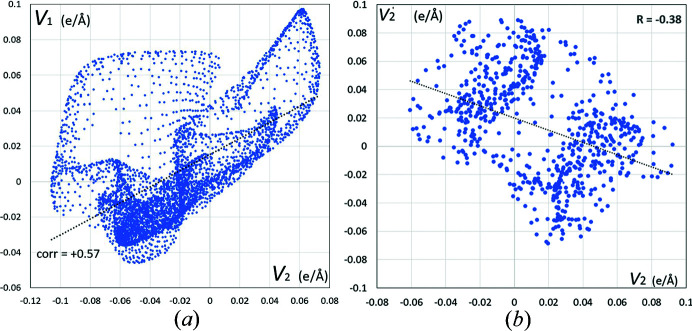
Scatterplot of the electrostatic potentials generated by two GA molecules involved in aromatic stacking interactions in the GA_4_PyW_4_ crystal. The points are obtained on the Hirshfeld surface (limited by ρ_tot_ > 0.001 e Å^−3^) between the two molecules. (*a*) *V*_1_(**r**) versus *V*_2_(**r**) in the parallel stacking dimer between GA1 and GA2 molecules, (*b*) *V*_2_′(**r**) versus *V*_2_(**r**) in the antiparallel stacking dimer between two GA2 molecules.

Furthermore, a scatterplot of deformation electrostatic potentials (*V*_1_,*V*_2_) generated by the GA molecules on the Hirshfeld surface of the two stacking interactions in GA_4_PyW_4_ is shown in Fig. 7 ▸. The plots are generated using *MoProViewer*. As expected, the parallel stacking does not show electrostatic complementarity as the cloud of points (*V*_1_,*V*_2_) is closer to the diagonal line *V*_1_ = *V*_2_ and even has a positive correlation, *R* = +0.57 [Fig. 7 ▸(*a*)]. The antiparallel stacking results in a scatterplot closer the diagonal line *V*_2_′ = −*V*_2_ and indicates a partial electrostatic complementarity with a negative correlation, *R* = −0.38 [Fig. 7 ▸(*b*)]. Antiparallel stacking is able to realize a partial electrostatic complementarity due to the presence of hetero atoms O besides C and H atoms (Salonen *et al.*, 2011 ▸; Jelsch *et al.*, 2014 ▸).

### 3.4. Experimental static and dynamic charge density analysis

#### 3.4.1. Global descriptors of the charge density distributions

**Figure 8:**
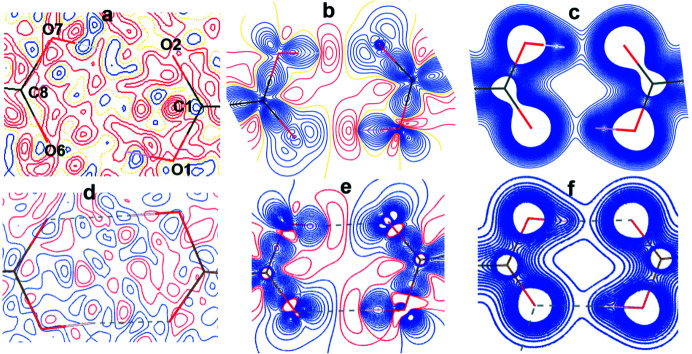
Electron density maps in the plane of the acid dimer (O1, O2, C8 plane) of the GA_4_PyW_4_ cocrystal: (*a*) residual, (*b*) static deformation and (*c*) static total density maps in the experimental MP model. (*d*), (*e*) and (*f*) are the residual, static deformation and static total density maps, respectively, from the XWR method at λ_max_ = 0.7. The contour levels are 0.05 e Å^−3^ for all density maps. For the total density, contours are at 0.2 e Å^−3^ up to 4.0 e Å^−3^ at an interval of 0.05 e Å^−3^. Blue and red solid lines indicate positive and negative electron density, respectively.

**Figure 9:**
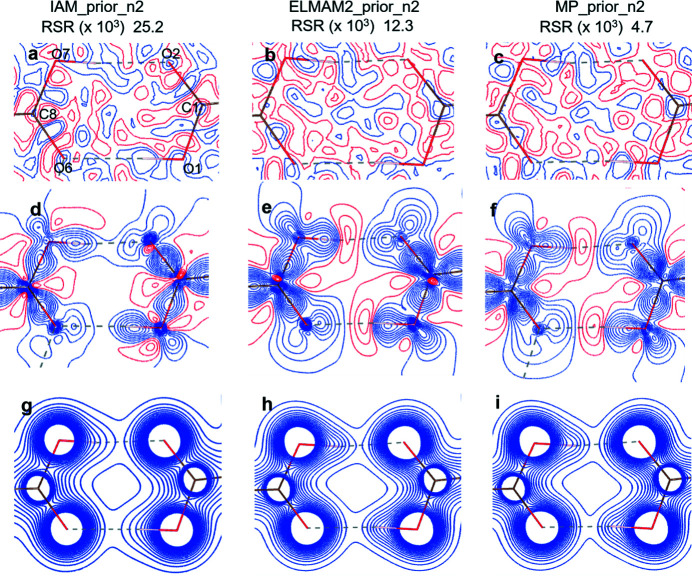
Dynamic electron density maps in the plane of the acid dimer (O1, O2, C8 plane) of the GA_4_PyW_4_ cocrystal: first, second and third columns represent 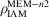 , 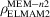 and 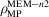 , respectively, (*a*), (*b*), (*c*) are residual density (difference Fourier maps), (*d*), (*e*), (*f*) are dynamic deformation density and (*g*), (*h*), (*i*) are dynamic total electron density. The contour level is at 0.05 e Å^−3^ for all maps. For the total density, contours are at 0.2 e Å^−3^ up to 4.0 e Å^−3^. Blue and red solid lines indicate positive and negative electron density, respectively. The RSR values are listed at the top for each case of 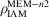 , 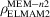 and 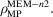

One of the major differences between static and dynamic densities is expected at the positions close to the locations of the atoms. Local maxima in the dynamic electron densities are not obtained for most H atoms and they appear as a ‘shoulder’ on the density of the atom to which it is covalently bonded (Hofmann *et al.*, 2007 ▸; Mondal *et al.*, 2012 ▸; Prathapa *et al.*, 2013 ▸). However, the non-H atom positions, covalent bonds and hydrogen bonds in molecular crystals are well described by dynamic model densities and MEM densities with various priors and these are generally on par with static MP density analysis. The local maxima for non-H atoms are found at nearly equal positions in different dynamic density maps as shown in Table S6. The residual, deformation and total electron density maps obtained for GA_4_PyW_4_ from static MP and XWR models are shown in Fig. 8 ▸. The dynamic MEM densities (weight *n* = 2) obtained with three different priors are shown in Fig. 9 ▸. A similar figure for MEM density with weighting scheme *n* = 4 is shown in Fig. S6.

The peaks are randomly distributed in all Fourier residual map cases in Figs. 8 ▸ and 9 ▸. The enhanced lobes of the lone pairs of the oxygen atoms are prominent for those which are involved in strong O—H⋯O hydrogen bonding compared to those which are not involved. To the best of our knowledge, it is the first ever direct comparison of XWR and MEM densities. The differences in the deformation density maps between XWR and MEM shows that larger differences are in the core region whereas the covalent bonding and intermolecular regions are similar in all methods.

The details of different types of MEM calculations, listed in Table 2 ▸, shown in Fig. 9 ▸, indicate that the spread of final *wR*_F_ values is much smaller compared to the initial ones. Hence, the resultant MEM densities obtained with different priors and different weighting schemes are closer to each other compared to the prior dynamic model densities. The MEM density differs the most from the prior density in the case of the IAM prior and it is closest for the MP prior.

The overall similarity between the charge distributions was examined quantitatively by the real space R values as shown in Fig. 9 ▸. Here, the MP prior produces the smallest RSR value, indicating that it is closest to the corresponding MEM density. The degree of similarity decreases in the following order MP-prior > ELMAM2-prior > IAM-prior. A previous study of MEM densities on amino acids and peptides for different prior densities indicated the superiority of MP priors (Prathapa *et al.*, 2013 ▸). Here, a similar trend is obtained that the MP-prior density is a better description of the electron density distribution compared to the IAM-prior density.

#### 3.4.2. Topological properties at covalent bonds

A quantitative analysis of the topological properties at bond critical points (BCPs) for the covalent bonds (C—O, C=O and C—C bonds) in the COOH group, phenolic and phenyl rings from the three dynamic model densities are listed in Table 7 ▸ and Tables S7 and S8, respectively. In agreement with a previous report (Prathapa *et al.*, 2013 ▸), the IAM density differs significantly from ELMAM2 and MP densities because it is promolecular. Overall, the trend is as follows: IAM << ELMAM2 ∼ MP. The polar C—O and C=O bonds in COOH and the phenolic COH group are especially poorly described with low ρ(**r**)_bcp_ and positive ∇^2^ρ(**r**)_bcp_ values in IAM. The ELMAM2 model density results in negative ∇^2^ρ(**r**)_bcp_ values for all the C—O and C=O polar bonds. However, the magnitudes of ρ(**r**)_bcp_ and ∇^2^ρ(**r**)_bcp_ values for C=O bonds are consistently lower in ELMAM2 than in the MP dynamic model densities. For the aromatic and aliphatic C—C bonds, ∇^2^ρ(**r**)_bcp_ values in the IAM dynamic model density are at least negative as expected for a covalent bond, but the magnitudes are much lower. Similarly, ρ(**r**)_bcp_ values are also lower. ELMAM2 and MP dynamic model densities produce similar values for aliphatic and aromatic C—C bonds and phenolic C—O bonds.

For the MEM analysis on GA_4_PyW_4_, the two weighting schemes lead to similar topological properties for the covalent bonds in case of individual prior densities. The results obtained from weight *n* = 4 in the MEM analysis with three different priors are listed in Table S9. The 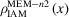 result improved the description of ρ(**r**_CP_) compared to 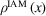 . The ∇^2^ρ(**r**_cp_) for polar C—O bonds turn out to be slightly negative, but the C=O bonds still indicate positive values. Overall, the trend remains as IAM << ELMAM2 ∼ MP.

The magnitudes of the values of ρ(**r**_cp_) and ∇^2^ρ(**r**_cp_) for the C—C bonds from the experimental static MP model and the XWR method are consistently and significantly larger compared to the dynamic models and the MEM electron densities. For the polar C—O and C=O bonds, the ∇^2^ρ(**r**_cp_) values approximately agree between the dynamic MEM results based on ELMAM2/MP and the static XWR results, but they are significantly too large for the static MP model. It was shown on a set of high-resolution X-ray diffraction datasets of amino acids and tripeptides [Fig. 5 ▸ in Woińska *et al.* (2017 ▸)] that the XWR model is superior over the multipole model especially for the accurate description of polar bonds such as C—O and C=O since for the amino acids and tripeptides the ∇^2^ρ(**r**_cp_) values were also too large sometimes by a factor of 2 or 3. The general trend is followed in static and dynamic densities that with symmetric bonds such as C—C, the bonding density accumulates in the middle of the bond regions where the CP is, whereas, for C—O and C=O bonds, the CP is more towards the lighter C atom. This finding agrees with Fig. 4 ▸ in Woińska *et al.* (2017 ▸). This means that the dynamic MEM densities are a more reliable alternative to free multipole modelling for the description of polar bonds.

Topological analysis on the gas phase calculations at B3LYP-D3/def2-TZVP and B3LYP-D3/6-311++g(2d,2p) level (last two columns in Table 7 ▸) indeed reveals that for the polar C—O and C=O bonds, the ∇^2^ρ(**r**_cp_) values are on par with MEM and XWR densities. Hence, they support the finding that ∇^2^ρ(**r**_cp_) values for these polar C—O and C=O bonds are too large only for the static MP density. It is noteworthy that the two basis set families, 6-311++g(2d,2p) and def2-TZVP, indeed influence the more sensitive ∇^2^ρ(**r**_cp_) values for the polar bonds, specifically the C=O bonds (−8.6 versus −15.5 e Å^−5^, respectively, for C1=O2, and −8.7 versus −15.5 e Å^−5^, respectively, for C8=O6).

#### 3.4.3. Topological properties of hydrogen bonds

The topological parameters for the hydrogen bonds forming the acid dimer are listed in Table 8 ▸. The two hydrogen bonds are not of equal strength. The O7−H7O⋯O2 bond with shorter O⋯O distance has slightly higher ρ(**r**_cp_) and ∇^2^ρ(**r**_cp_) values than the O1−H1⋯O6 bond. Thus, the hydrogen bond energy, *E*_HB_, which is defined as derived from the local potential energy density (Espinosa *et al.*, 1998 ▸) is ∼25 kJ mol^−1^ higher for the O7−H7O⋯O2 bond. In contrast to the covalent bonds, for hydrogen bonds the IAM dynamic model density and MEM density with IAM prior have almost always slightly higher ρ(**r**_cp_) values compared to other dynamic or static densities. The relative ratio of local kinetic and potential energy densities, |*V*_CP_|/*G*_CP_, is listed for both the hydrogen bonds. |*V*_CP_|/*G*_CP_ > 1 indicates that the interaction is stabilized by a local concentration of the charge (Espinosa *et al.*, 2002 ▸). For the stronger O7−H7O⋯O2 hydrogen bond, |*V*_CP_|/*G*_CP_ > 1 is obtained for all static and dynamic densities. For the relatively weaker O1−H1⋯O6 hydrogen bond, the ratio varies, depending on the electron density model. The ratio |*V*_CP_|/*G*_CP_ is higher in the stronger O7−H7O⋯O2 hydrogen bond compared to the weaker O1−H1⋯O6 for a given electron density model, which follows the general trend reported earlier (Espinosa *et al.*, 2002 ▸). The topological properties of the hydrogen bonds and the hydrogen bond energies for the two water environments obtained from the static density in the experimental multipole model are listed in Table S10.

#### 3.4.4. The π-hole carbon bonding interaction

**Figure 10:**
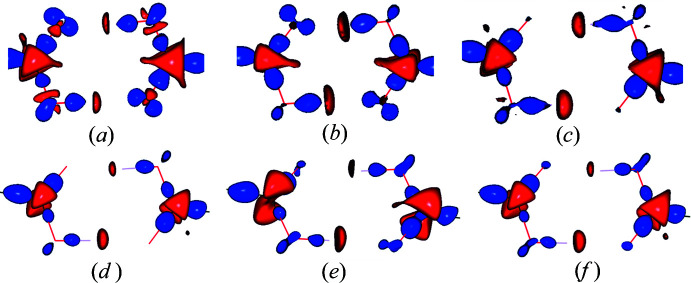
(*a*, *b*, *c*) Three-dimensional static deformation density obtained from XWR (XCW at λ_max_ = 0.7), ELMAM2 database transfer model, experimental multipole model, respectively. (*d*, *e*, *f*) Three-dimensional dynamic deformation density representing 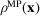 , 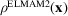 , 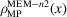 , respectively. The isocontour level is +0.3/−0.15 e Å^−3^. The blue and red regions indicate positive and negative deformation electron density, respectively.

The 3D static deformation density from the experimental multipole model, the ELMAM2 database transferred model and XWR are shown in Fig. 10 ▸ along with the 3D dynamic deformation density maps from the dynamic model density with the multipole prior, the MEM density (MP prior, weight *n =* 2) and the MEM density (ELMAM2 prior, weight *n* = 2). Both the carb­oxy­lic C atoms, C1 and C8, display electron deficient regions above and below the molecular plane (red colour) which is a signature of a π-hole. In the XWR and ELMAM2 static density maps, there is a small electron deficient region on the carbonyl O atom also (O2 and O6), although it is much smaller compared to the carb­oxy­lic C atoms (C1 and C8).

**Figure 11:**
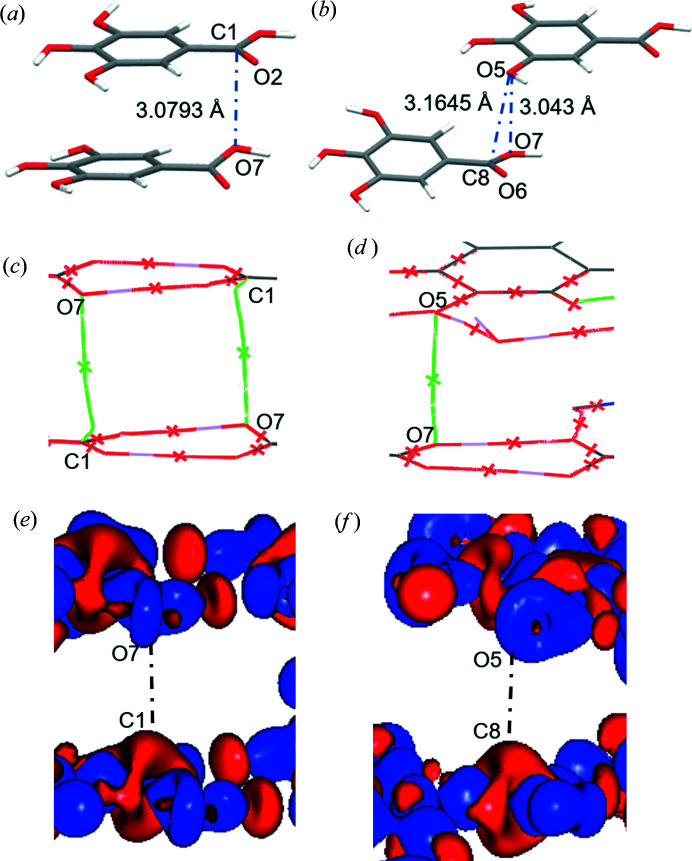
Parallel stacking in (*a*) dimer1// and (*b*) dimer3. The distances for C1⋯O7, C8⋯O5 and O5⋯O7 contacts are shown. Molecular graphs obtained from topological analysis of experimental dynamic density from MEM analysis, 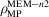 for (*c*) dimer1// and (*d*) dimer3. (The geometry is from experimental MP model.) The crosses depict the positions of the bond critical points. (*e*, *f*) Three-dimensional experimental static deformation density obtained from multipole model for the two stacking dimers. Colour code: blue (positive), red (negative)

Three different dimers are considered in the overall study. Table 9 ▸ shows the geometry (bond distance and angles) of the concerned dimers and Fig. 5 ▸ shows their arrangement graphically. Dimer1// and dimer2_anti// represent the two types of aromatic stacking interactions, parallel and antiparallel. Dimer1// and dimer3 represent the π-holes on the carb­oxy­lic C atoms. Both C1 and C8 are involved in π-hole carbon bonding interactions with neighbouring electron-rich O atoms [Figs. 11 ▸(*a*) and 11 ▸(*b*)]. The π-hole on C1 interacts with the neighbouring electronegative carb­oxy­lic O7 atom forming the π-hole interaction C1⋯O7. The COOH group in GA2 (with C8 as carb­oxy­lic C) is slightly deviated from the plane of the phenyl ring. This arrangement made the π-hole interaction C1⋯O7 favourable (dimer1//), with *d*(C1⋯O7) = 3.0793 (5) Å and ∠O2=C1⋯O7 = 85.01 (2)°, close to ∼90°, appropriate for a π-hole bonding. The same conformation rearrangement of GA2 that makes C1⋯O7 favourable has also led C8 away and O7 closer to hydroxyl O5 of neighbouring GA1. The O7⋯O5 contact (*d*_OO_ = 3.0430 Å) is therefore slightly shorter than C8⋯O5 (*d*_CO_ = 3.1645 Å) (dimer3). This means that dimer3 is involved in the shortest contact with a neighbouring electron-rich O atom.

The complementarity of the static deformation density maps, issued from the multipolar model, within the parallel stacking dimer1// and within the dimer3 is shown in Figs. 11 ▸(*e*) and 11 ▸(*f*). In dimer1//, the electron deficient carboxyl C1 of the COOH group is facing towards the electron-rich site of hydroxyl O7 in COOH, resulting in π-hole bonding. In dimer3, the electron deficient carboxyl C8 of the COOH group is facing towards the electron-rich site of phenolic O5 atom, although the topological analysis does not produce a C8⋯O5 bond path, see below.

**Figure 12:**
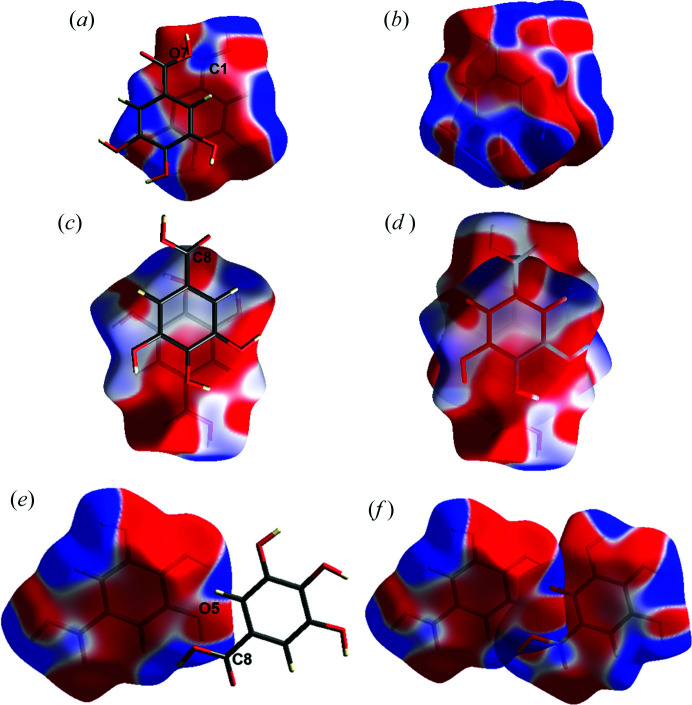
Electrostatic potential (ESP) mapped onto molecular Hirshfeld surfaces for the three dimers; dimer1// in (*a*,*b*); dimer2_anti// (*c*,*d*) and dimer3 (*e*,*f*). The colour scale is −0.01 au (red) to 0 au (white) to 0.1 au (blue).

Static electrostatic potential maps obtained from the *Crystal Explorer* calculation at B3LYP/6-31g(d,p) level are shown in Fig. 12 ▸. They describe the electrostatic complementarity in all three dimers. Concerning the π-hole interactions, they show electropositive regions at the carboxyl C atoms, C1 and C8, and how they face the electronegative regions at the oxygen atoms. There is a more pronounced electrostatic complementarity in dimer2_anti// concerning π-stacking (see also Fig. 7 ▸).

The bond path of the C1⋯O7 interaction from the 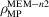 density is shown in Fig. 11 ▸(*c*). However, there was no bond path obtained for the C8⋯O5 contact. The bond path was found for the O7⋯O5 contact instead [Fig. 11 ▸(*d*)]. The details of topological properties of the C1⋯O7 and O7⋯O5 interactions are listed in Table 10 ▸ obtained from different electron density models. The π-hole (C1⋯O7) was identified ubiquitously in the static density in the experimental MP model and in all the three dynamic model densities. Between the adjacent acid dimers, two such C1⋯O7 interactions are expected. For MEM densities, only 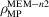 yielded the bond path and the corresponding bond critical point for the C1⋯O7 interaction [Fig. 11 ▸(*c*)]. However, there was no bond path obtained for the C8⋯O5 contact in any of the models, but instead a bond path for the O7⋯O5 contact for all the dynamic and static densities [Fig. 11 ▸(*d*)]. It is worth noting that bond paths related to weak interactions fluctuate a lot between atoms concerned when the electron density model is changed. They are very much affected by experimental and model errors. For example, none of the MEM calculations with the IAM prior could locate a bond path for the C1⋯O7 interaction. In case of the ELMAM2 prior, with *n* = 4 weighting scheme, 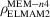 , bond paths were identified for C1⋯O7 and O2⋯O7 contacts (Table S11). The magnitudes of ρ(**r**)_bcp_ in the dynamic densities both in MEM results and prior densities are slightly larger than those from the static density in the experimental multipole model, as reported earlier (Hofmann *et al.*, 2007 ▸; Prathapa *et al.*, 2013 ▸). The relative strength of this closed-shell interaction based on the ratio of the local potential energy density to the kinetic energy density (|*V*_bcp_|/*G*_bcp_) derived from the ρ(**r**_cp_) and ∇^2^ρ(**r**_cp_) values is also noted down in Table 10 ▸ and the ratio is found to be less than 1, as reported for another π-hole bonding, Br⋯C(π) (Shukla *et al.*, 2018 ▸). This study demonstrates that it is difficult to identify the weak π-hole interactions in the electron-density distributions in terms of topological parameters, however, some bond paths could be identified in the dynamic MEM density and their description is on par with the static multipole model.

These difficulties in identifying bond paths and bond critical points for the specific atom–atom contacts in the π-hole interactions indicate that intermolecular interactions in these dimers must be viewed in a more wholistic fashion (Dunitz, 2015 ▸). The calculated interaction energies at B3LYP/6-31g(d,p) level in *Crystal Explorer*, for dimer1//, dimer2_anti// and dimer3 are −20.7, −30.9 and −14.2 kJ mol^−1^, respectively (Table 9 ▸), stabilizing the overall crystal packing. The breakdown of the total energy into its components shows that it is the dispersion energy component, and not the small electrostatic component, that clearly dominates the dimer interactions. Although there is more electrostatic complementarity in the antiparallel dimer (Fig. 7 ▸), leading to more stabilization via electrostatics according to Table 9 ▸, the complementarity of charge concentration/depletion in these interactions as shown in Figs. 11 ▸ and 12 ▸ does not manifest itself as electrostatically generated atom–atom interactions unlike strong hydrogen bonds. Gavezzotti (2013 ▸) states, as already cited and discussed by Edwards *et al.* (2017 ▸) ‘In many cases, with the exception of hydrogen bonding, molecular pairings responsible for the largest part of the interaction energy in a crystal show no particular atom–atom feature, no easily identifiable ‘bond’, not even aromatic stacks, or the like; they stick together by compatibility of minor and diffuse features in the electrostatic potential, that defy recognition and, a fortiori, classification’. However, the interactions within both dimers may still be called a π-hole carbon bonding interaction as the arrangement of the diffuse electrostatic potential seems to be governed by the carb­oxy­lic C atom depletions and O atoms concentrations across some area of the molecular surfaces. To deal with such difficulty to classify elusive intermolecular interactions, Alhameedi *et al.* (2018 ▸) introduced and investigated ‘bond orders for intermolecular interactions’, taking into account molecule⋯molecule instead of atom⋯atom interactions. Such bond orders would include orbital interactions and charge transfer, which are not separately captured in the *Crystal Explorer* model energies. In the next subsection, we therefore consider orbital interactions and charge transfer in more detail using natural bond orbital analysis, which exceeds a single bond order.

#### 3.4.5. Natural bond orbital analysis

**Figure 13:**
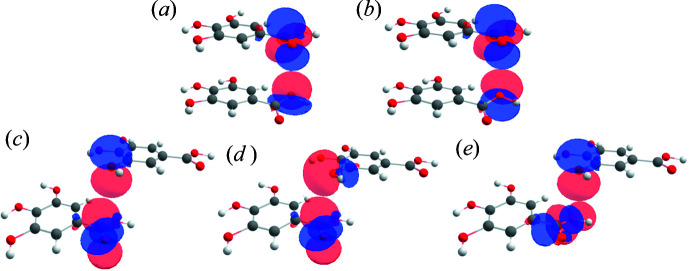
(*a*, *b*) Inter-orbital interactions from NBO analysis between two lone pairs of O7 with π*(C1=O2) in dimer1//. (*c*, *d*) Inter-orbital interactions from NBO analysis between two lone pairs of O5 with π*(C8=O6) in dimer3. Blue and red represent the opposite signs of the orbitals.

The NBO analysis of the two dimers, dimer1// and dimer3, establishes the inter-orbital interactions involving charge transfer from lone pairs of O atoms to the π*(C=O) orbitals corresponding to the π-hole containing C1 and C8 atoms (Table 11 ▸, Fig. 13 ▸). For dimer1//, there is an occurrence of charge transfer from the two lone pairs of O7, *i.e.* O7(lp1) and O7(lp2), to the π*(C1=O2) orbital with the second-order perturbation energies *E*(2), 0.79 and 1.09 kcal mol^−1^, respectively. Thus, the total magnitude of *E*(2) for the O7(lp)→π*(C1=O2) inter-orbital interaction is 1.88 kJ mol^−1^. Similarly, in the case of dimer3, the charge transfer from the two lone pairs of O5, *i.e.* O5(lp1) and O5(lp2), to the π*(C8=O6) orbital attributes *E*(2) values of 0.54 and 1.00 kJ mol^−1^ to the total interaction energy. Thus, the total magnitude of the charge transfer interactions corresponds to 1.55 kJ mol^−1^, which is not far from that of dimer1//. Hence, both the carb­oxy­lic C atoms, C1 and C8 with π-holes, get stability from the charge transfer from lone pairs of neighbouring O atoms, but the related energies are quite small. This means that the NBO analysis corroborates the viewpoint discussed above that π-hole bonding cannot be seen as an interaction that is energetically dominated by a directed atom–atom contact.

## 4. Conclusions

In the new cocrystal hydrate of gallic acid with pyrazine, GA_4_PyW_4_, the two independent gallic acid molecules of the asymmetric unit adopt *syn* COOH conformation and form the most common acid dimer synthon. The crystal structure is primarily stabilized by strong O—H⋯O hydrogen bonds and aromatic stacking interactions. The GA2 conformation, which has two internal hydrogen bonds within the three phenol groups, is more stable *in vacuo* compared to GA1, but in the crystal state, GA1 gains more stability from the interactions with neighbouring molecules. In the crystal state, the molecular dipole moment of GA1 gets increased, whereas the one for GA2 slightly decreases compared to *in vacuo*. The dimeric interaction energy for antiparallel stacking of GA1⋯GA1 is more negative compared to the parallel stacking of GA1⋯GA2. Also, the antiparallel stacking of GA1⋯GA1 results in an attractive (negative) electrostatic contribution and shows partial electrostatic complementarity.

There is an electron deficient region above and below the molecular plane that includes the carb­oxy­lic C1 and C8 atoms indicating the signature of π-holes. It was identified and examined by experimental static and dynamic electron density analysis. The COOH group in GA2 shows a small deviation from the plane of the phenyl ring which slightly destabilizes this conformer. This deviation in GA2, however, brings the O7 atom of COOH closer to the carboxyl C1 atom of the neighbouring acid dimer and thus favours the formation of a π-hole carbon bonding interaction between adjacent layers. Both the static density descriptions in MP and XWR methods as well as the dynamic density in the MEM approach unequivocally describe the signature of π-hole interactions in the acid dimers. However, the π-hole interactions cannot be pinned down to individual atom–atom intermolecular contacts, but the dimer interactions are dominated by dispersion forces and some charge transfer identified by NBO analysis, whereas electrostatic contributions to these dimers are negligible.

From a methodological point of view, this study demonstrates that similar to the covalent and hydrogen bonds, the relatively weaker π-hole interactions could be identified in the dynamic MEM density and the description is on par with the static multipole model. Furthermore, our study presents the first comparison of the XWR and MEM methods of experimental charge density analysis. With data of good quality such as for GA_4_PyW_4_ used here, qualitative features of electron-density distributions are identical, whereas topological values and intermolecular interaction energies are overestimated in MP relative to XWR and MEM, especially for polar bonds such as C—O and C=O.

## Supplementary Material

Crystal structure: contains datablock(s) I. DOI: 10.1107/S2052520622001457/px5047sup1.cif

Structure factors: contains datablock(s) I. DOI: 10.1107/S2052520622001457/px5047Isup2.hkl

Tables S1 to S11 and Figs S1 to S6. DOI: 10.1107/S2052520622001457/px5047sup3.pdf

CCDC reference: 2112784

## Figures and Tables

**Table:** 

Chemical formula	4(C_7_H_6_O_5_)(C_4_H_4_N_2_)˙4(H_2_O)
*M*_r_ (g mol^−1^	832.60
Crystal system, space group	Triclinic, *P* 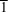
Temperature (K)	100
*a*, *b*, *c* (Å)	7.7242 (8), 9.0042 (1), 13.3769 (1)
α, β, γ (°)	92.440 (4), 96.223 (4), 112.469 (3)
*V* (Å^3^)	851.09 (9)
*Z*	1
Density (g cm^−3^)	1.625
*F*(000)	434
Radiation type	Mo *K*α
μ (mm^−1^)	0.14
Crystal size (mm)	0.25 × 0.15 × 0.13

(sin θ/λ)_max_ (Å^−1^)	1.00

θ_min_, θ_max_ (°)	2.5, 45.3
Max resolution (Å)	0.50
No. of reflections	118 595
No. of unique reflections	14 250
*R*_int_	0.041

**Table:** 

Refinement	IAM	Multipolar	XWR†
Program	*SHELXL*	*MoPro*	*TONTO*
Weighting scheme	‡	*w* = 1.76/σ^2^(*I*_o_)	*w* = 1/σ(*F*_o_)
No. of reflections [*F* > 4σ(*F*)]	11 776	11 776	11 776
*R*_F_	0.0404	0.0228	0.0255
*wR* ^2^ *I*	0.1339	0.0287	0.0342
Δρ_min_, Δρ_max_ (e Å^−3^)	−0.80, 0.98	−0.29, 0.24	−0.27, 0.25

The refinement details for MEM analysis are listed in Table 2 ▸.

† XCW fitting up to a λ value of 0.7 is considered.

‡ Weighting scheme in *SHELXL*: *w* = 1/[σ^2^(*F*_o_^2^) + (a*P*)^2^ + *bP*] , where *P* is [2*F*_c_^2^ + Max(*F*_o_^2^,0)]/3.

**Table 2:** Details of MEM calculations The number of pixels is 156 × 182 × 268 in all cases, which leads to a step size of 0.04973 Å.

	IAM prior		ELMAM2 prior		Multipole prior	
Weighting scheme	*n* = 2	*n* = 4	*n* = 2	*n* = 4	*n* = 2	*n* = 4
No. of reflections [*F* > 3σ(*F*)]	12 179	12 179	12 179	12 179	12 179	12 179
Initial *R*_F_, *wR*_F_	0.0349, 0.0410	0.0349, 0.0410	0.0302, 0.0268	0.0302, 0.0268	0.0253, 0.0156	0.0253, 0.0156
Final *R*_F_, *wR*_F_	0.0228, 0.0145	0.0231, 0.0145	0.0245, 0.0148	0.0247, 0.0148	0.0246, 0.0148	0.0247, 0.0148
Δρ (min, max) (e Å^−3^)	−0.27, 0.24	−0.30, 0.26	−0.24, 0.30	−0.26, 0.30	−0.27, 0.27	−0.25, 0.28

**Table 3:** Figures of merit for different HARs The basis set is def2-TZVP for all.

	HAR(RHF)	HAR(BLYP)	HAR(B3LYP)
χ^2^	2.7104	2.1703	2.0790
*R*(*F*)	0.0259	0.0245	0.0243
*wR*(*F*)	0.0169	0.0151	0.0148
Δρ_min_, Δρ_max_ (e Å^−3^)	−0.305, 0.251	−0.295, 0.239	−0.287, 0.239

**Table 4:** Flexible torsion angles (in °) in the two symmetry-independent gallic acid molecules, GA1 and GA2, obtained from multipole and XWR refinements of the crystal structure and from the geometry optimization in the isolated state

	Crystal geometry	Multipole	HAR (RHF)	HAR (BLYP)	HAR (B3LYP)	Gaussian opt
GA1	φ_1_(H1—O1—C1—C2)	178.1 (3)	178.7 (4)	178.4 (3)	178.5 (3)	180.01
GA2	φ_1_(H7O—O7—C8—C9)	174.2 (3)	173.7 (3)	174.3 (3)	174.2 (3)	180.01
GA1	φ_2_(C7—C2—C1—O2)	1.69 (4)	1.70 (4)	1.67 (4)	1.69 (3)	0.02
GA2	φ_2_(C10—C9—C8—O6)	−14.94 (4)	−14.95 (4)	−14.93 (3)	−14.93 (3)	0.09
GA1	φ_3_(H3O—O3—C4—C5)	161.4 (4)	161.3 (4)	161.2 (3)	161.2 (3)	180.03
GA2	φ_3_(H10O—O10—C13—C12)	16.9 (4)	16.1 (4)	16.3 (3)	16.3 (3)	0.06
GA1	φ_4_(H4—O4—C5—C6)	−8.1 (3)	−9.2 (3)	−8.8 (3)	−8.9 (3)	−0.10
GA2	φ_4_(H9—O9—C12—C11)	0.4 (3)	0.6 (4)	0.2 (3)	0.2 (3)	0.23
GA1	φ_5_(H5—O5—C6—C7)	−5.4 (3)	−5.8 (3)	−5.4 (3)	−5.4 (3)	−0.06
GA2	φ_5_(H8—O8—C1—C10)	−0.8 (3)	−1.4 (3)	−0.9 (3)	−1.0 (3)	−0.13

**Table 5:** Cohesive energy (kJ mol^−1^) based on periodic calculations using the *CRYSTAL14* package at B3LYP-D2/pob-TZVP_2012 level

	Δ*E*_cond_	Δ*E*_conf_	BSSE	Δ*E*_cohesive_
GA1	−207.2	18.9	62.5	−125.9
GA2	−189.9	19.6	75.8	−94.5

**Table 6:** Analysis of contacts on the Hirshfeld surface of all moieties in the GA_4_PyW_4_ cocrystal Reciprocal contacts *X*⋯*Y* and *Y*⋯*X* are merged. The % of contact types between chemical species is given followed by their enrichment ratio. The second line shows the chemical contents on the surface. The major contacts as well as the major enriched ones are highlighted in bold. The hydro­phobic hydrogen atoms bound to carbon (Hc) were distinguished from the more polar ones bound to oxygen (Ho). The last three rows show the contacts analysis in terms of grouped hydro­phobic (HPB: Hc and C) and hydro­philic (HPL: O, N, Ho) atoms.

Atom	O	N	Ho	Hc	C
Surface (%)	29.9	3.1	27.4	14.4	25.3
O	2.7				
N	0.4	0		% Actual contacts	
Ho	**33.1**	3	4.5		
Hc	**8.7**	0.5	7.4	2.6	
C	**11.1**	0.6	6.6	5.9	**13**
O	0.31				
N	0.28	0		Enrichment	
Ho	**1.91**	**2.26**	0.52		
Hc	1.08	0.75	0.9	**1.36**	
C	0.75	0.58	0.45	0.84	**2.07**
Surface (%)	HPL	60.4	HPB 39.6		
Contacts (%)	HPL	43.6	HPB 21.5	(HPL, HPB)	34.9
Enrichment (*E*)	HPL	1.20	HPB 1.36	(HPL, HPB)	0.73

**Table 7:** Topological properties of covalent bonds of the COOH group in GA_4_PyW_4_ ρ(**r**)_bcp_ (e Å^−3^; first line) and ∇^2^ρ(**r**)_bcp_ (e Å^−5^; second line) for the ten different electron density distributions: three dynamic model densities, three MEM densities with *n*_2_ weighting scheme, four static densities; two of them correspond to experimental MP model and XWR and the last two columns represent gas phase calculations using the B3LYP-D3 method with two different basis sets, def2-TZVP and 6-311++G(2d,2p), respectively

	Dynamic model density			MEM (*n*_2_)			Static			
	IAM	ELMAM2	MP	IAM	ELMAM2	MP	MP	XWR	B3LYP-D3/ def2-TZVP	B3LYP-D3/6-311++G(2d,2p)
C1—O1	1.80	2.34	2.19	2.00	2.19	2.17	2.34	2.26	2.03	2.02
	6.4	−17.7	−13.9	−1.4	−14.2	−13.7	−27.6	−17.5	−18.3	−18.5
C1=O2	2.08	2.59	2.71	2.44	2.68	2.71	2.89	2.78	2.90	2.88
	20.8	−5.9	−14.5	18.2	−8.9	−15.3	−37.2	−9.4	−8.6	−15.5
C8—O7	1.81	2.37	2.24	2.03	2.26	2.22	2.37	2.29	2.05	2.03
	6.5	−18.9	−15.8	−1.1	−16.9	−15.9	−28.6	−18.0	−18.4	−18.6
C8=O6	2.08	2.60	2.72	2.42	2.64	2.70	2.87	2.76	2.90	2.88
	20.5	−7.2	−16.9	14.9	−10.9	−17.2	−36.7	−15.1	−8.7	−15.5
C1—C2	1.27	1.77	1.78	1.67	1.73	1.76	1.88	1.89	1.88	1.83
	−1.3	−13.5	−14.1	−15.0	−12.6	−13.5	−15.1	−18.9	−18.9	−16.3
C8—C9	1.27	1.78	1.78	1.72	1.77	1.77	1.86	1.91	1.88	1.82
	−1.4	−13.6	−13.8	−18.9	−15.2	−14.6	−14.5	−19.8	−18.9	−16.2

**Table 8:** Topological properties at the CPs of the hydrogen bonds forming the GA1⋯GA2 acid dimer: H⋯O and O⋯O distances (Å), O—H⋯O angle (°), electron density (e Å^−3^), Laplacian (e Å^−5^) at bond critical points, local potential over kinetic energy densities at bond critical points and hydrogen-bond dissociation energy (kJ mol^−1^) XWR geometry is HAR(B3LYP) geometry; for MEM with IAM prior, the geometry is IAM geometry after *SHELXL* refinement; for MEM with ELMAM2 and MP prior, the geometry is experimental MP model geometry.

		Dynamic model density			MEM(*n*2)			Static density	
		IAM	ELMAM2	MP	IAM	ELMAM2	MP	MP	XWR
O7—H7O⋯O2	H⋯O	1.69 (2)	1.585 (5)	1.585 (5)	1.69 (2)	1.585 (5)	1.585 (5)	1.585 (5)	1.604 (6)
	O⋯O	2.5942 (6)	2.5949 (4)	2.5949 (4)	2.5942 (6)	2.5949 (4)	2.5949 (4)	2.5949 (4)	2.5942 (3)
	∠O—H⋯O	171 (1)	172.7 (5)	172.7 (5)	171 (1)	172.7 (5)	172.7 (5)	172.7 (5)	174.3 (5)
	ρ	0.38	0.39	0.34	0.38	0.39	0.34	0.34	0.39
	∇^2^ρ	2.5	2.1	4.7	2.0	1.7	4.5	5.5	3.1
	|*V*|/*G*	1.39	1.44	1.08	1.45	1.51	1.10	1.02	1.28
	*E*_HB_	−74.8	−73.5	−73.5	−72.2	−73.5	−73.5	−77.5	−77.5
O1—H1⋯O6	H⋯O	1.69 (2)	1.692 (5)	1.692 (5)	1.69 (2)	1.692 (5)	1.692 (5)	1.692 (5)	1.728 (6)
	O⋯O	2.7070 (6)	2.7067 (4)	2.7067 (4)	2.7070 (6)	2.7067 (4)	2.7067 (4)	2.7067 (4)	2.7062 (3)
	∠O—H⋯O	177 (2)	176.0 (5)	176.0 (5)	177 (2)	176.0 (5)	176.0 (5)	176.0 (5)	178.2 (5)
	ρ	0.31	0.31	0.25	0.31	0.30	0.25	0.24	0.28
	∇^2^ρ	2.0	1.7	4.2	1.7	1.8	4.2	4.7	2.8
	|*V*|/*G*	1.29	1.38	0.93	1.39	1.31	0.95	0.89	1.15
	*E*_HB_	−52.51	−52.5	−49.9	−51.2	−49.9	−49.9	−51.2	−51.2

**Table 9:** The interaction energy (kJ mol^−1^) obtained from *Crystal Explorer* for the three dimers Only the π-hole bonding interactions are listed for dimer1// and dimer3. (The aromatic stacking interaction details are not listed, so there are no geometric entries for dimer2.). See also Fig. 11 ▸.

Dimer	π-Hole interaction separation (Å)	π-Hole interaction angle (°)	*E*_ele	*E*_pol	*E*_dis	*E*_rep	*E*_tot
1 //	C1⋯O7: 3.0793 (5)	∠O2=C1⋯O7: 85.01 (2)	0.4	−1.8	−45.1	31.6	−20.7
	O2⋯O7: 3.2168 (5)	∠C1=O2⋯O7: 72.48 (2)					
2_anti//	No π-hole, but π-stacking		−9.2	−1.4	−46.1	32.3	−30.9
3	C8⋯O5: 3.1645 (5)	∠O6=C8⋯O5: 97.30 (2)	−4.8	−0.7	−15.2	7.5	−14.2
	O7⋯O5: 3.0430 (5)	∠C8=O7⋯O5: 82.99 (2)					

**Table 10:** Topological properties of C1⋯O7 (π-hole carbon bonding) and O7⋯O5 interactions are listed The MEM densities which found O2⋯O7 bond paths instead of C1⋯O7 are listed in brackets. There are no XWR results for the π-hole interaction because only the asymmetric unit was used in the XCW fitting procedure.

		Dynamic model density			MEM (*n* = 2)			Static
		IAM	ELMAM2	MP	IAM	ELMAM2	MP	MP
C1⋯O7	*R*_*ij*_ (Å)	3.0927	3.2590	3.0000	(3.5525)	(3.4158)	3.3027	3.2191
(O2⋯O7)	ρ (e Å^−3^)	0.063	0.051	0.054	(0.050)	(0.048)	0.051	0.046
	∇^2^ρ (e Å^−5^)	0.81	0.64	0.66	(0.61)	(0.52)	0.62	0.62
	|*V*|/*G*	0.71	0.80	0.80	(0.80)	(0.75)	0.80	0.80
O7⋯O5	*R*_*ij*_ (Å)	3.0810	3.0585	3.0568	3.0789	3.0823	3.0586	3.0429
	ρ (e Å^−3^)	0.059	0.058	0.058	0.060	0.057	0.057	0.049
	∇^2^ρ (e Å^−5^)	0.86	0.806	0.84	0.88	0.85	0.86	0.75
	|*V*|/*G*	0.71	0.71	0.71	0.71	0.71	0.71	0.69

**Table 11:** Second-order perturbation energy *E*(2) in kJ mol^−1^ for the C⋯O interactions

Dimer	Orbitals involved	*E*(2)
1 //	O7(lp1)→π*(C1=O2)	0.79
	O7(lp2)→π*(C1=O2)	1.09
3	O5(lp1)→π*(C8=O6)	0.54
	O5(lp2)→π*(C8=O6)	1.00
