# Supplementary material for: π-Hole bonding in a new co-crystal hydrate of gallic acid and pyrazine: static and dynamic charge density analysis
Source: Acta Crystallogr B Struct Sci Cryst Eng Mater. 2022 Mar 23;78(Pt 2):231–46. doi: 10.1107/S2052520622001457 (PMC9004022; doi:10.1107/S2052520622001457)
Supplement: Supplementary file 3 [file b-78-00231-sup3.pdf]

STRUCTURAL SCIENCE
CRYSTAL ENGINEERING
MATERIALS

Volume 78 (2022)

Supporting information for article:

π-Hole bonding in a new co-crystal hydrate of gallic acid and pyrazine: static and dynamic charge density analysis

Rumpa Pal, Christian Jelsch, Koichi Momma and Simon Grabowsky

Table S1. The details of XCW fitting procedure is shown. The values of χ^2 at each λ step and corresponding electronic energies are listed.

λ	χ^2	E (Hartree)	$\Delta E=E(\lambda_i)-E(\lambda_0)$
0	3.09	-1701.26	0.00
0.05	2.52	-1701.25	29.59
0.1	2.30	-1701.23	71.70
0.15	2.17	-1701.22	112.62
0.2	2.08	-1701.2	150.86
0.25	2.02	-1701.19	186.52
0.3	1.98	-1701.18	219.93
0.35	1.94	-1701.16	251.57
0.4	1.91	-1701.15	281.49
0.45	1.88	-1701.14	310.05
0.5	1.86	-1701.13	337.44
0.55	1.84	-1701.12	363.81
0.6	1.83	-1701.11	389.30
0.65	1.81	-1701.1	414.01
0.7	1.80	-1701.09	438.04

Table S2. The H-bond geometries of the phenolic OH groups in Gal1 and Gal2.

D–H···A		D–H (Å)	H···A (Å)	D···A (Å)	D–H···A(°)	Symmetry
Gal1						
O3–H3O···O6	Multipole	0.976(5)	1.864(5)	2.7938(4)	158.1(5)	x,-1+y,z
	HAR(RHF)	0.997(5)	1.841(5)	2.7939(4)	158.8(5)	
	HAR(BLYP)	0.953(5)	1.883(5)	2.7946(3)	159.3(5)	
	HAR(B3LYP)	0.962(4)	1.874(5)	2.7945(3)	159.2(4)	
O10–H10O···O3	Multipole	0.976(3)	1.937(3)	2.8485(4)	154.5(5)	1-x,-y,-z
	HAR(RHF)	1.005(6)	1.907(5)	2.8493(4)	154.9(5)	
	HAR(BLYP)	0.958(6)	1.950(5)	2.8495(4)	155.3(5)	
	HAR(B3LYP)	0.968(4)	1.941(4)	2.8495(4)	155.3(4)	
O4–H4···O8	Multipole	0.977(4)	1.887(5)	2.7127(4)	140.4(4)	2-x,1-y,-z
	HAR(RHF)	0.998(5)	1.869(5)	2.7131(3)	140.4(5)	
	HAR(BLYP)	0.956(4)	1.899(4)	2.7135(3)	141.6(4)	
	HAR(B3LYP)	0.965(4)	1.892(4)	2.7135(3)	141.3(4)	

O11–H11B···O4	Multipole	0.980(6)	1.900(6)	2.8718(5)	170.8(5)	-x+1,-y, z+1
	HAR(RHF)	0.982(7)	1.901(7)	2.8711(4)	169.3(5)	
	HAR(BLYP)	0.948(6)	1.935(6)	2.8714(4)	168.9(5)	
	HAR(B3LYP)	0.955(6)	1.928(6)	2.8713(4)	168.9(5)	
O9–H9···O5	Multipole	0.976(4)	2.051(5)	2.8987(4)	144.0(4)	2-x,1-y,-z
	HAR(RHF)	0.986(5)	2.030(6)	2.8996(3)	145.8(5)	
	HAR(BLYP)	0.947(4)	2.072(4)	2.9001(3)	145.2(4)	
	HAR(B3LYP)	0.955(4)	2.063(4)	2.9000(3)	145.3(4)	
O5–H5···O12	Multipole	0.977(4)	1.648(3)	2.6085(4)	166.9(5)	2-x,1-y,1-z
	HAR(RHF)	1.037(5)	1.589(5)	2.6097(4)	166.8(5)	
	HAR(BLYP)	0.970(5)	1.652(5)	2.6094(4)	168.5(5)	
	HAR(B3LYP)	0.984(5)	1.639(5)	2.6095(4)	168.1(5)	
Gal2						
O8–H8···O11	Multipole	0.977(4)	1.697(3)	2.6673(3)	171.6(5)	2-x,1-y,1-z
	HAR(RHF)	1.019(5)	1.654(5)	2.6678(3)	172.1(5)	
	HAR(BLYP)	0.964(5)	1.708(5)	2.6675(3)	173.0(5)	
	HAR(B3LYP)	0.976(4)	1.696(4)	2.6675(3)	172.8(5)	
O4–H4···O8	Multipole	0.977(4)	1.887(5)	2.7127(4)	140.4(4)	2-x,1-y,-z
	HAR(RHF)	0.998(5)	1.869(5)	2.7131(3)	140.4(5)	
	HAR(BLYP)	0.956(4)	1.899(4)	2.7135(3)	141.6(4)	
	HAR(B3LYP)	0.965(4)	1.892(4)	2.7135(3)	141.3(4)	
O9–H9···O5	Multipole	0.976(4)	2.051(5)	2.8987(4)	144.0(4)	2-x,1-y,-z
	HAR(RHF)	0.986(5)	2.030(6)	2.8996(3)	145.8(5)	
	HAR(BLYP)	0.947(4)	2.072(4)	2.9001(3)	145.2(4)	
	HAR(B3LYP)	0.955(4)	2.063(4)	2.9000(3)	145.3(4)	
O10–H10O···O3	Multipole	0.976(3)	1.937(3)	2.8485(4)	154.5(5)	1-x,-y,-z
	HAR(RHF)	1.005(6)	1.907(5)	2.8493(4)	154.9(5)	
	HAR(BLYP)	0.958(6)	1.950(5)	2.8495(4)	155.3(5)	
	HAR(B3LYP)	0.968(4)	1.941(4)	2.8495(4)	155.3(4)	
O11–H11A···O10	Multipole	0.979(5)	1.825(5)	2.7967(4)	171.4(5)	1-x,-y,1-z
	HAR(RHF)	0.987(6)	1.819(5)	2.7975(4)	170.6(5)	
	HAR(BLYP)	0.961(5)	1.845(5)	2.7971(4)	170.7(5)	

	HAR(B3LYP)	0.966(5)	1.840(5)	2.7972(3)	170.6(5)	

Table S3. The Geometrical parameters for Gal1 obtained from experimental multipole model, three types of HAR methods and the optimized geometry *in vacuo*.

	Crystal Geometry	Multipole	HAR (RHF)	HAR (BLYP)	HAR (B3LYP)	Gaussian opt
Torsion	φ1(H1-O1-C1-C2)	178.1(3)	178.7(4)	178.4(3)	178.5(3)	-179.99
	φ2(C7-C2-C1-O2)	1.69(4)	1.70(4)	1.67 (4)	1.69(3)	0.02
	φ3(H3O-O3-C4-C5)	161.4(4)	161.3(4)	161.2(3)	161.2(3)	-179.97
	φ4(H4-O4-C5-C6)	-8.1(3)	-9.2(3)	-8.8(3)	-8.9(3)	-0.10
	φ5(H5-O5-C6-C7)	-5.4(3)	-5.8(3)	-5.4(3)	-5.4(3)	-0.06
Bond	θ1(H1-O1-C1)	106.8(3)	108.2(5)	108.0(6)	108.1(5)	106.36
angle	θ2(H3O-O3-C4)	109.7(3)	112.6(6)	111.8(5)	112.0(5)	109.47
	θ3(H4-O4-C5)	108.6(3)	108.3(5)	108.8(5)	108.7(5)	108.46
	θ4(H5-O5-C6)	111.0(3)	110.8(5)	111.6(5)	111.5(5)	110.29
Bond	O1-H1	1.016(5)	1.018(6)	0.969(6)	0.979(6)	0.97
length	O3-H3O	0.976(5)	0.997(5)	0.953(5)	0.962(4)	0.96
	O4-H4	0.977(4)	0.998(5)	0.956(4)	0.965(4)	0.96
	O5-H5	0.977(4)	1.037(5)	0.970(5)	0.984(5)	0.96
	C1-O1	1.3178(4)	1.3159(3)	1.3166(3)	1.3163(3)	
	C1=O2	1.2365(4)	1.2325(3)	1.2323(3)	1.2322(3)	
	C4-O3	1.3675(4)	1.3657(3)	1.3662(3)	1.3659(3)	1.36
	C5-O4	1.3612(4)	1.3601(4)	1.3607(3)	1.3604(3)	1.35
	C6-O5	1.3591(4)	1.3570(3)	1.3573(3)	1.3571(3)	1.38

Table S4. The Geometrical parameters for Gal2 obtained from experimental multipole model, three types of HAR methods and the optimized geometry *in vacuo*.

Torsion	φ1(H7O-O7-C8-C9)	174.2(3)	173.7(3)	174.3(3)	174.2(3)	-179.99
	φ2(C10-C9-C8-O6)	-14.94(4)	-14.95(4)	-14.93(3)	-14.93(3)	0.09
	φ3(H10O-O10-C13-C12)	16.9(4)	16.1(4)	16.3(3)	16.3(3)	0.06

	φ4(H9-O9-C12-C11)	0.4(3)	0.6(4)	0.2(3)	0.2(3)	0.23
	φ5(H8-O8-C11-C10)	-0.8(3)	-1.4(3)	-0.9(3)	-1.0(3)	-0.13
Bond	θ1(H7O-O7-C8)	107.3(3)	108.5(5)	108.3(5)	108.3(5)	106.43
angle	θ2(H10O-O10-C13)	110.5(3)	112.7(5)	112.3(5)	112.4(5)	109.08
	θ3(H9-O9-C12)	107.7(3)	108.8(5)	108.0(5)	108.2(5)	109.17
	θ4(H8-O8-C11)	110.1(3)	109.9(5)	110.2(5)	110.1(5)	110.25
Bond	O7-H7O	1.015(5)	1.037(6)	0.983(6)	0.994(6)	0.97
length	O10-H10O	0.976(3)	1.005(6)	0.958(6)	0.968(4)	0.96
	O9-H9	0.976(4)	0.986(5)	0.947(4)	0.955(4)	0.96
	O8-H8	0.977(4)	1.019(5)	0.964(5)	0.976(4)	0.96
	C8-O7	1.3164(4)	1.3149(3)	1.3154(3)	1.3152(3)	
	C8=O6	1.2428(4)	1.2392(3)	1.2396(3)	1.2393(3)	
	C13-O10	1.3674(3)	1.3663(3)	1.3668(3)	1.3665(3)	1.36
	C12-O9	1.3561(4)	1.3552(3)	1.3556(3)	1.3554(3)	1.37
	C11-O8	1.3640(4)	1.3626(3)	1.3632(3)	1.3630(3)	1.38
	O11-H11A	0.979(5)	0.987(6)	0.961(5)	0.966(5)	0.96
	O11-H11B	0.980(6)	0.982(7)	0.948(6)	0.955(6)	0.96
	O12-H12A	0.981(6)	0.997(7)	0.958(6)	0.966(6)	0.96
	O12-H12B	0.979(5)	0.972(6)	0.934(6)	0.942(5)	0.96
	H11A-O11-H11B	103.5(4)	105.3(8)	105.9(8)	105.9(8)	105.05
	H12A-O12-H12B	103.7(4)	107.2(8)	107.0(8)	107.2(8)	105.14

Table S5. The H-bond geometries for the acid dimeric motifs as obtained from three types of HAR refinements and multipole model.

D–H···A		D–H (Å)	H···A (Å)	D···A (Å)	D–H···A(°)	Symmetry
O1–H1···O6	Multipole	1.016(5)	1.692(5)	2.7067(4)	176.0(5)	X ; Y ; Z
	HAR(RHF)	1.018(6)	1.688(6)	2.7055(4)	178.2(5)	
	HAR(BLYP)	0.969(6)	1.738(6)	2.7064(3)	178.0(5)	
	HAR(B3LYP)	0.979(6)	1.728(6)	2.7062(3)	178.2(5)	
O7–H7O···O2	Multipole	1.015(5)	1.585(5)	2.5949(4)	172.7(5)	X ; Y ; Z
	HAR(RHF)	1.037(6)	1.559(6)	2.5934(4)	174.7(5)	
	HAR(BLYP)	0.983(6)	1.615(6)	2.5944(3)	174.2(5)	

	HAR(B3LYP)	0.994(6)	1.604(6)	2.5942(3)	174.3(5)	

Table S6. Coordinates of atomic maxima in nine different density maps of Ga_2PyW_2: Dynamic model densities and MEM density maps for IAM (first line), ELMAM2(second line), MP (third line).

	Dynamic model density			MEM density(weight n2)			MEM density(weight n4)		
	x	y	z	x	y	z	x	y	z
O1	0.5356	0.2010	0.5114	0.5356	0.2010	0.5114	0.5356	0.2010	0.5114
	0.5356	0.2011	0.5115	0.5356	0.2011	0.5115	0.5356	0.2011	0.5115
	0.5356	0.2011	0.5115	0.5356	0.2011	0.5115	0.5356	0.2011	0.5115
O2	0.7260	0.4613	0.5137	0.7260	0.4613	0.5137	0.7260	0.4613	0.5137
	0.726	0.4613	0.5137	0.726	0.4613	0.5137	0.726	0.4613	0.5137
	0.726	0.4613	0.5137	0.726	0.4613	0.5137	0.726	0.4613	0.5137
O4	0.9310	0.1752	0.1249	0.9310	0.1752	0.1249	0.9310	0.1752	0.1249
	0.9310	0.1754	0.1249	0.9310	0.1754	0.1249	0.9310	0.1754	0.1249
	0.9310	0.1754	0.1249	0.9310	0.1754	0.1249	0.9310	0.1754	0.1249
O5	1.0737	0.4822	0.2109	1.0737	0.4822	0.2109	1.0737	0.4822	0.2109
	1.0737	0.4822	0.2109	1.0737	0.4822	0.2109	1.0737	0.4822	0.2109
	1.0737	0.4822	0.2109	1.0737	0.4822	0.2109	1.0737	0.4822	0.2109
C1	0.6639	0.3213	0.4740	0.6639	0.3213	0.4740	0.6639	0.3213	0.4740
	0.6639	0.3211	0.4740	0.6639	0.3211	0.4740	0.6639	0.3211	0.4740
	0.6639	0.3211	0.4740	0.6639	0.3211	0.4740	0.6639	0.3211	0.4740
C2	0.7303	0.2788	0.3819	0.7303	0.2788	0.3819	0.7303	0.2788	0.3819
	0.7303	0.2789	0.3820	0.7303	0.2789	0.3820	0.7303	0.2789	0.3820
	0.7303	0.2789	0.3820	0.7303	0.2789	0.3820	0.7303	0.2789	0.3820
C3	0.6543	0.1210	0.3352	0.6543	0.1210	0.3352	0.6543	0.1210	0.3352
	0.6544	0.1210	0.3352	0.6544	0.1210	0.3352	0.6544	0.1210	0.3352
	0.6544	0.1210	0.3352	0.6544	0.1210	0.3352	0.6544	0.1210	0.3352
C4	0.7231	0.0884	0.2484	0.7231	0.0884	0.2484	0.7231	0.0884	0.2484
	0.7231	0.0882	0.2484	0.7231	0.0882	0.2484	0.7231	0.0882	0.2484
	0.7231	0.0882	0.2484	0.7231	0.0882	0.2484	0.7231	0.0882	0.2484
C5	0.8654	0.2120	0.2092	0.8654	0.2120	0.2092	0.8654	0.2120	0.2092
	0.8655	0.2119	0.2091	0.8655	0.2119	0.2091	0.8655	0.2119	0.2091
	0.8655	0.2119	0.2091	0.8655	0.2119	0.2091	0.8655	0.2119	0.2091
C6	0.9382	0.3701	0.2555	0.9382	0.3701	0.2555	0.9382	0.3701	0.2555
	0.9383	0.3701	0.2555	0.9383	0.3701	0.2555	0.9383	0.3701	0.2555
	0.9383	0.3701	0.2555	0.9383	0.3701	0.2555	0.9383	0.3701	0.2555
C7	0.8709	0.4032	0.3423	0.8709	0.4032	0.3423	0.8709	0.4032	0.3423
	0.8709	0.4033	0.3423	0.8709	0.4033	0.3423	0.8709	0.4033	0.3423
	0.8709	0.4033	0.3423	0.8709	0.4033	0.3423	0.8709	0.4033	0.3423

Gal2									
O6	0.4475	0.3150	0.6810	0.4475	0.3150	0.6810	0.4475	0.3150	0.6810
	0.4475	0.3150	0.6810	0.4475	0.3150	0.6810	0.4475	0.3150	0.6810
	0.4475	0.3150	0.6810	0.4475	0.3150	0.6810	0.4475	0.3150	0.6810
O7	0.6103	0.5758	0.6623	0.6103	0.5758	0.6623	0.6103	0.5758	0.6623
	0.6103	0.5758	0.6623	0.6103	0.5758	0.6623	0.6103	0.5758	0.6623
	0.6103	0.5758	0.6623	0.6103	0.5758	0.6623	0.6103	0.5758	0.6623
O8	0.1028	0.3615	0.9819	0.1028	0.3615	0.9819	0.1028	0.3615	0.9819
	0.1029	0.3615	0.9818	0.1029	0.3615	0.9818	0.1029	0.3615	0.9818
	0.1029	0.3615	0.9818	0.1029	0.3615	0.9818	0.1029	0.3615	0.9818
O9	0.3058	0.6685	1.0680	0.3058	0.6685	1.0680	0.3058	0.6685	1.0680
	0.3058	0.6685	1.0680	0.3058	0.6685	1.0680	0.3058	0.6685	1.0680
	0.3058	0.6685	1.0680	0.3058	0.6685	1.0680	0.3058	0.6685	1.0680
O10	0.6027	0.8748	0.9820	0.6027	0.8748	0.9820	0.6027	0.8748	0.9820
	0.6025	0.8747	0.9820	0.6025	0.8747	0.9820	0.6025	0.8747	0.9820
	0.6025	0.8747	0.9820	0.6025	0.8747	0.9820	0.6025	0.8747	0.9820
C8	0.5017	0.4598	0.7114	0.5017	0.4598	0.7114	0.5017	0.4598	0.7114
	0.5017	0.4599	0.7115	0.5017	0.4599	0.7115	0.5017	0.4599	0.7115
	0.5017	0.4599	0.7115	0.5017	0.4599	0.7115	0.5017	0.4599	0.7115
C9	0.4487	0.5151	0.8044	0.4487	0.5151	0.8044	0.4487	0.5151	0.8044
	0.4488	0.5151	0.8044	0.4488	0.5151	0.8044	0.4488	0.5151	0.8044
	0.4488	0.5151	0.8044	0.4488	0.5151	0.8044	0.4488	0.5151	0.8044
C10	0.2996	0.4067	0.8480	0.2996	0.4067	0.8480	0.2996	0.4067	0.8480
	0.2996	0.4067	0.8480	0.2996	0.4067	0.8480	0.2996	0.4067	0.8480
	0.2996	0.4067	0.8480	0.2996	0.4067	0.8480	0.2996	0.4067	0.8480
C11	0.2495	0.4577	0.9356	0.2495	0.4577	0.9356	0.2495	0.4577	0.9356
	0.2495	0.4577	0.9357	0.2495	0.4577	0.9357	0.2495	0.4577	0.9357
	0.2495	0.4577	0.9357	0.2495	0.4577	0.9357	0.2495	0.4577	0.9357
C12	0.3492	0.6151	0.9814	0.3492	0.6151	0.9814	0.3492	0.6151	0.9814
	0.3491	0.6152	0.9815	0.3491	0.6152	0.9815	0.3491	0.6152	0.9815
	0.3491	0.6152	0.9815	0.3491	0.6152	0.9815	0.3491	0.6152	0.9815
C13	0.5002	0.7214	0.9380	0.5002	0.7214	0.9380	0.5002	0.7214	0.9380
	0.5003	0.7216	0.9380	0.5003	0.7216	0.9380	0.5003	0.7216	0.9380
	0.5003	0.7216	0.9380	0.5003	0.7216	0.9380	0.5003	0.7216	0.9380
C14	0.5485	0.6729	0.8487	0.5485	0.6729	0.8487	0.5485	0.6729	0.8487
	0.5484	0.6729	0.8487	0.5484	0.6729	0.8487	0.5484	0.6729	0.8487
	0.5484	0.6729	0.8487	0.5484	0.6729	0.8487	0.5484	0.6729	0.8487

Table S7. Topological properties of aromatic C–C bonds of the phenyl rings in Gal1 and Gal2 in GPW: ρ_{BCP} (eÅ^{-3}; first line) and $\nabla^2\rho_{BCP}$ (eÅ^{-5}; second line) for the 11 different density maps (3

dynamic model densities, 6 MEM densities with two different weighting schemes and 2 static densities).

	dynamic model density			MEM(n2)			MEM(n4)			Static	
	IAM	ELMAM2	MP	IAM	ELMAM 2	MP	IAM	ELMAM2	MP	MP	XWR
C2-C3	1.44	1.96	2.00	1.93	1.99	1.99	1.91	1.96	1.97	2.08	2.13
	-3.4	-16.6	-16.8	-24.2	-19.1	-17.5	-23.0	-17.8	-17.0	-17.1	-23.7
C3-C4	1.44	2.06	2.02	1.92	2.00	2.00	1.91	1.99	1.99	2.14	2.16
	-3.3	-18.5	-17.1	-19.8	-16.4	-15.9	-18.8	-16.4	-15.7	-18.6	-22.6
C4-C5	1.43	2.10	2.04	1.94	2.03	2.02	1.94	2.01	2.01	2.14	2.17
	-3.3	-19.4	-18.2	-21.8	-17.0	-17.4	-22.2	-17.4	-17.4	-19.1	-23.2
C5-C6	1.44	2.12	2.06	1.98	2.06	2.05	1.97	2.04	2.03	2.15	2.19
	-3.4	-20.1	-19.1	-25.5	-20.2	-19.3	-24.4	-19.7	-19.1	-19.6	-25.1
C6-C7	1.46	2.09	2.04	1.91	2.02	2.02	1.92	2.01	2.02	2.14	2.15
	-3.5	-19.1	-17.5	-17.6	-16.7	-16.7	-17.9	-17.1	-16.8	-18.6	-22.0
C7-C2	1.44	1.96	1.97	1.90	1.96	1.96	1.89	1.95	1.95	2.06	2.10
	-3.3	-16.2	-16.0	-21.4	-16.3	-15.8	-20.3	-16.4	-15.9	-16.8	-21.4
C9-C10	1.45	1.97	1.98	1.90	1.99	1.98	1.89	1.97	1.96	2.06	2.12
	-3.4	-16.4	-16.0	-20.9	-17.3	-16.7	-20.4	-17.0	-16.2	-16.7	-22.4
C10-C11	1.47	2.10	2.06	1.97	2.05	2.05	1.96	2.03	2.04	2.15	2.19
	-3.6	-19.5	-18.0	-22.9	-19.0	-18.6	-22.3	-18.8	-18.4	-18.7	-24.0
C11-C12	1.44	2.13	2.06	1.96	2.05	2.04	1.95	2.03	2.02	2.13	2.18
	-3.4	-20.0	-18.7	-23.7	-18.9	-18.4	-23.1	-18.6	-18.1	-18.9	-24.2
C12-C13	1.44	2.11	2.04	1.94	2.04	2.01	1.92	2.02	1.99	2.13	2.16
	-3.3	-19.6	-17.5	-24.0	-18.9	-17.0	-22.3	-18.1	-16.7	-18.4	-23.3
C13-C14	1.46	2.08	2.03	1.92	2.01	2.03	1.92	2.00	2.01	2.14	2.15
	-3.5	-19.0	-17.7	-20.5	-18.0	-17.8	-20.3	-17.9	-17.6	-18.7	-22.5
C14-C9	1.45	1.97	2.00	1.92	2.00	2.00	1.91	1.97	1.98	2.08	2.14
	-3.5	-16.9	-16.8	-21.7	-17.2	-17.1	-21.3	-17.0	-16.8	-16.9	-23.1

Table S8. Topological properties of the phenolic C–O bonds in Gal1 and Gal2 in GPW: ρ_{BCP} (eÅ^{-3}; first line) and $\nabla^2\rho_{BCP}$ (eÅ^{-5}; second line) for the 11 different density maps (3 dynamic model densities, 6 MEM densities with two different weighting schemes and 2 static densities).

	dynamic model density			MEM(n2)			MEM(n4)			Static	
	IAM	ELMAM2	MP	IAM	ELMAM2	MP	IAM	ELMAM2	MP	MP	XWR
C5-O4	1.66	1.94	1.90	1.75	1.88	1.89	1.76	1.87	1.88	2.03	1.96
	2.5	-11.4	-12.2	-4.4	-10.1	-11.5	-5.2	-10.5	-11.8	-16.7	-12.2
C11-O8	1.66	1.95	1.91	1.77	1.91	1.90	1.77	1.90	1.89	2.02	1.99

	2.5	-11.7	-11.8	-4.4	-11.2	-11.5	-4.8	-11.4	-11.4	-16.5	-15.6
C13-O10	1.65	1.93	1.93	1.79	1.89	1.91	1.78	1.87	1.90	2.05	1.99
	3.0	-10.6	-12.4	-9.4	-12.3	-12.6	-8.2	-11.4	-12.5	-16.3	-14.8

Table S9. Topological properties of covalent bonds of the –COOH group in GA_2PyW_2: $\rho(\mathbf{r})_{bcp}$ (eÅ^{-3}; first line) and $\nabla^2\rho(\mathbf{r})_{bcp}$ (eÅ^{-5}; second line) for the 3 MEM densities with n4 weighting scheme.

	MEM (*n*=4)		
	IAM	ELMAM2	MP
C1-O1	1.99<br>-1.2	2.18<br>-14.5	2.16<br>-13.9
C1=O2	2.42<br>17.6	2.65<br>-8.5	2.70<br>-15.6
C8-O7	2.01<br>-0.5	2.24<br>-17.2	2.21<br>-16.1
C8=O6	2.40<br>15.2	2.63<br>-10.4	2.69<br>-17.7
C1-C2	1.67<br>-15.4	1.73<br>-13.0	1.74<br>-13.0
C8-C9	1.72<br>-18.6	1.75<br>-14.9	1.75<br>-14.5

Table S10. The H-bond geometries for the two water environments obtained from multipole model and three types of HAR methods.

D–H···A		D–H (Å)	H···A (Å)	D···A (Å)	D–H···A(°)	Symmetry
O11–H11A···O10	Multipole	0.979(5)	1.825(5)	2.7967(4)	171.4(5)	X-1 ; Y-1 ; Z
	HAR(RHF)	0.987(6)	1.819(5)	2.7975(4)	170.6(5)	
	HAR(BLYP)	0.961(5)	1.845(5)	2.7971(4)	170.7(5)	
	HAR(B3LYP)	0.966(5)	1.840(5)	2.7972(3)	170.6(5)	
O11–H11B···O4	Multipole	0.980(6)	1.900(6)	2.8718(5)	170.8(5)	-x+1;-y; z+1
	HAR(RHF)	0.982(7)	1.901(7)	2.8711(4)	169.3(5)	
	HAR(BLYP)	0.948(6)	1.935(6)	2.8714(4)	168.9(5)	

	HAR(B3LYP)	0.955(6)	1.928(6)	2.8713(4)	168.9(5)	
O8–H8···O11	Multipole	0.977(4)	1.697(3)	2.6673(3)	171.6(5)	X ; Y ; Z
	HAR(RHF)	1.019(5)	1.654(5)	2.6678(3)	172.1(5)	
	HAR(BLYP)	0.964(5)	1.708(5)	2.6675(3)	173.0(5)	
	HAR(B3LYP)	0.976(4)	1.696(4)	2.6675(3)	172.8(5)	
O12–H12B···O11	Multipole	0.979(5)	1.906(5)	2.8699(4)	167.7(5)	-X+1 ; -Y+1 ; -Z+1
	HAR(RHF)	0.972(6)	1.913(6)	2.8692(4)	167.5(5)	
	HAR(BLYP)	0.934(6)	1.952(6)	2.8694(4)	167.2(5)	
	HAR(B3LYP)	0.942(5)	1.943(5)	2.8694(4)	167.3(4)	
O12–H12A···N1	Multipole	0.981(6)	1.815(6)	2.7934(5)	174.8(5)	X ; Y ; Z
	HAR(RHF)	0.997(7)	1.801(7)	2.7923(5)	172.8(6)	
	HAR(BLYP)	0.958(6)	1.839(6)	2.7929(4)	173.6(5)	
	HAR(B3LYP)	0.966(6)	1.832(6)	2.7930(4)	173.2(5)	
O5–H5···O12	Multipole	0.977(4)	1.648(3)	2.6085(4)	166.9(5)	X ; Y ; Z
	HAR(RHF)	1.037(5)	1.589(5)	2.6097(4)	166.8(5)	
	HAR(BLYP)	0.970(5)	1.652(5)	2.6094(4)	168.5(5)	
	HAR(B3LYP)	0.984(5)	1.639(5)	2.6095(4)	168.1(5)	

Table S11. Topological properties of C1···O7 (π-hole bonding) and O5···O7 interactions are listed for MEM densities with weight , n=4. The MEM densities which found O2···O7 bond paths instead of C1···O7 are listed in brackets.

		MEM(n4)		
		IAM	ELMAM2	MP
C1···O7	R_{ij}(Å)	-	3.1898	(3.4682)
(O2···O7)	ρ (eÅ^{-3})	-	0.046	(0.049)
	$\nabla^2\rho$ (eÅ^{-5})	-	0.63	(0.60)
	$\|V\|/G$		0.80	(0.80)
O5···O7	R_{ij}(Å)	3.0603	3.0608	3.0586
	ρ (eÅ^{-3})	0.063	0.059	0.059
	$\nabla^2\rho$ (eÅ^{-5})	0.98	0.92	0.88

	$\lvert V\rvert/G$	0.75	0.71	0.71

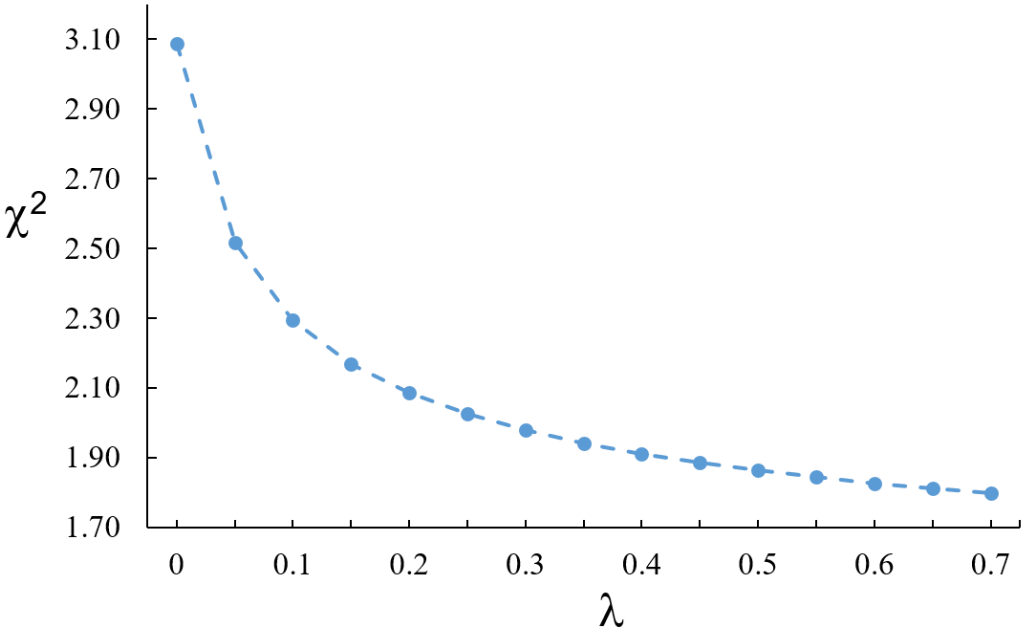

Figure S1. The χ^2 agreement statistic for the XCW fitting as a function of the Lagrange fitting parameter, λ.

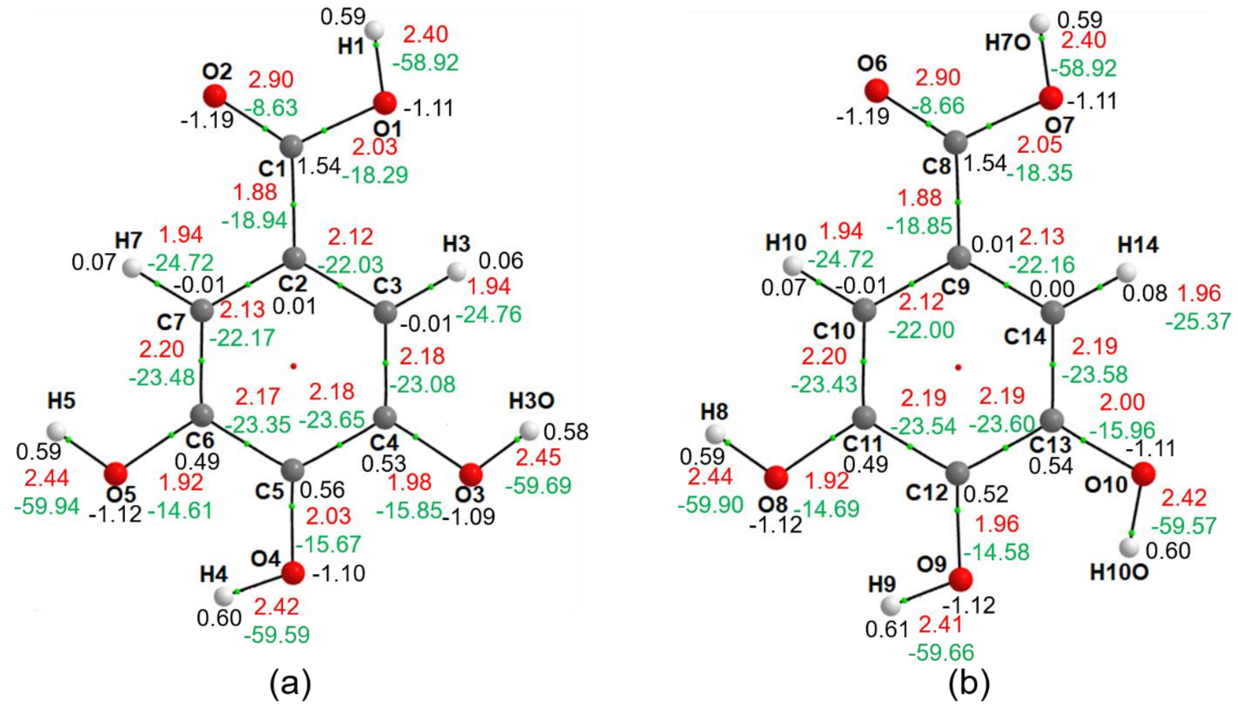

Figure S2. Molecular graphs of (a) GA1 (with *conf4*) and (b) GA2 (with *conf2*), obtained from AIMALL based on optimized geometries *in vacuo* in Gaussian16. The bond critical points (bcps) are shown in green circles. The Bader atomic charge of all the atoms are shown in black. The electron density [$\rho(\mathbf{r})$ (e Å^{-3})], Laplacian [$\nabla^2\rho(\mathbf{r})$ (e Å^{-5})] values at bcps are shown in red and green, respectively.

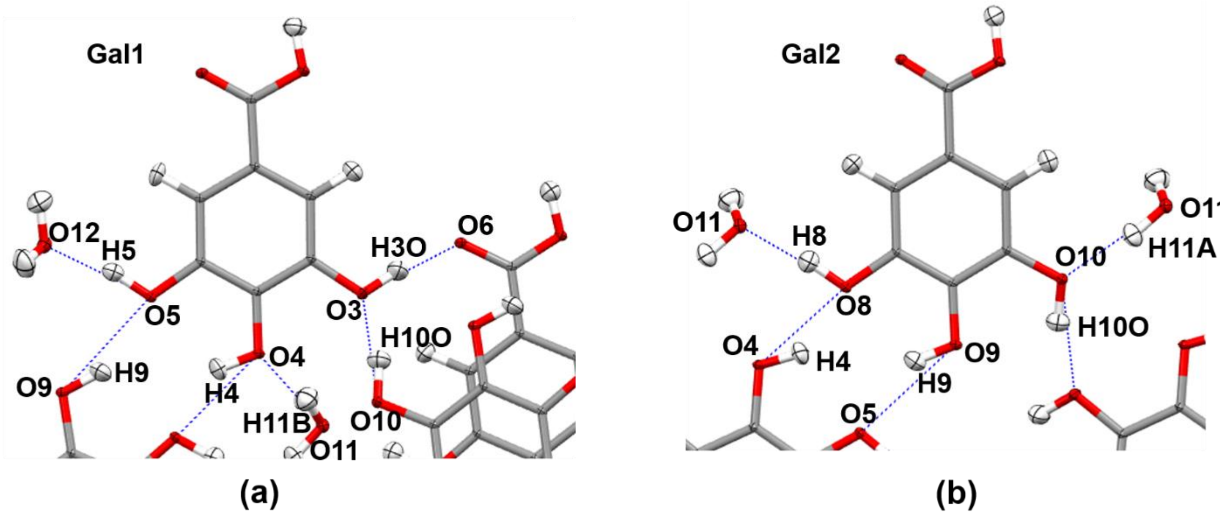

Figure S3. (a) The phenolic OH groups in Gal1 forms a total of six H-bonds with neighboring molecules (b) the phenolic OH groups in Gal2 form a total of five intermolecular H-bonds.

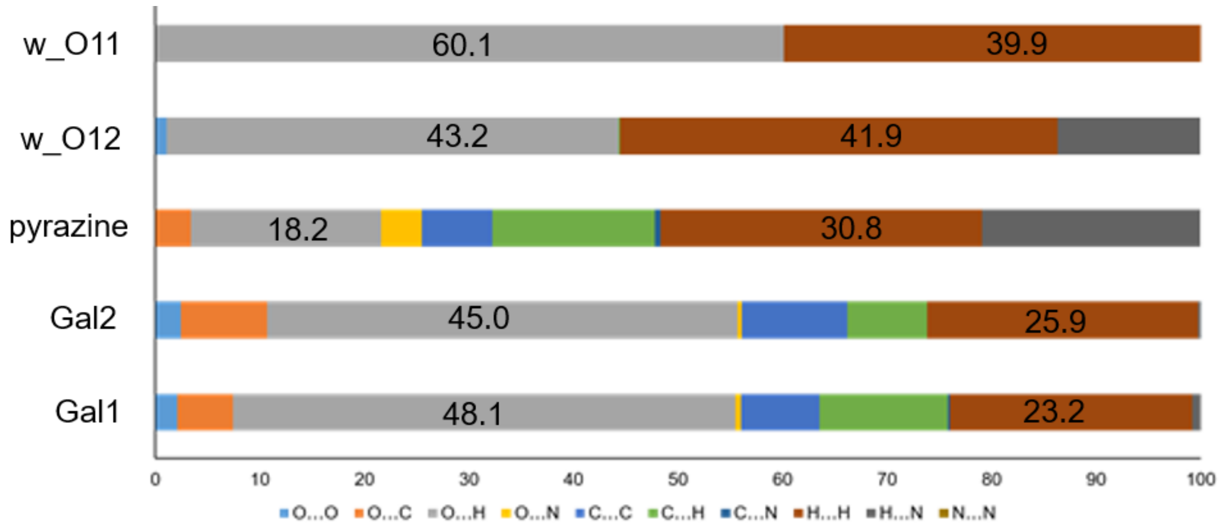

Figure S4. Relative contributions to the Hirshfeld surface area for various close intermolecular contacts obtained from Crystal Explorer.

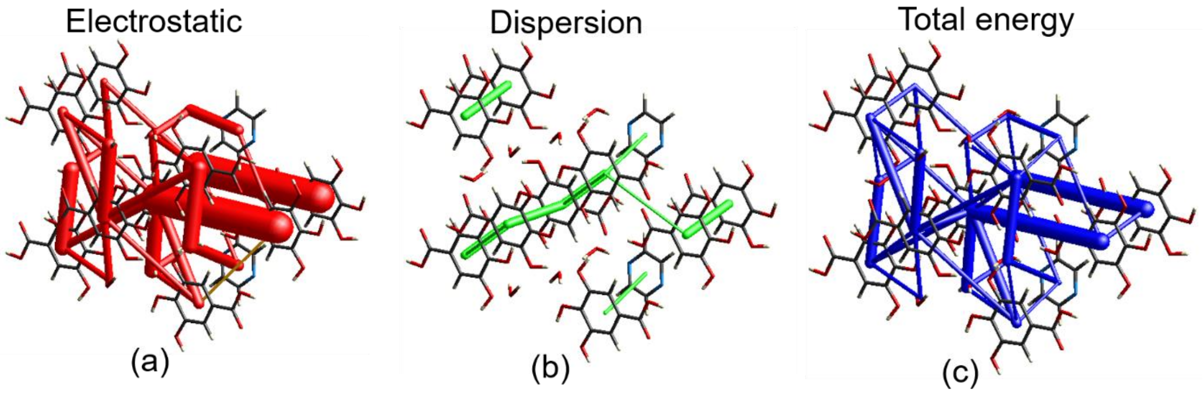

Figure S5. Energy frameworks for separate (a) electrostatic (red), (b) dispersion (green) contributions to the (c) total nearest pairwise interaction energies (blue) in GA2PyW2, viewed down *a* axis. The energy scale factor is 70, and interaction energies with magnitudes smaller than 12 kJ mol^{-1} are omitted.

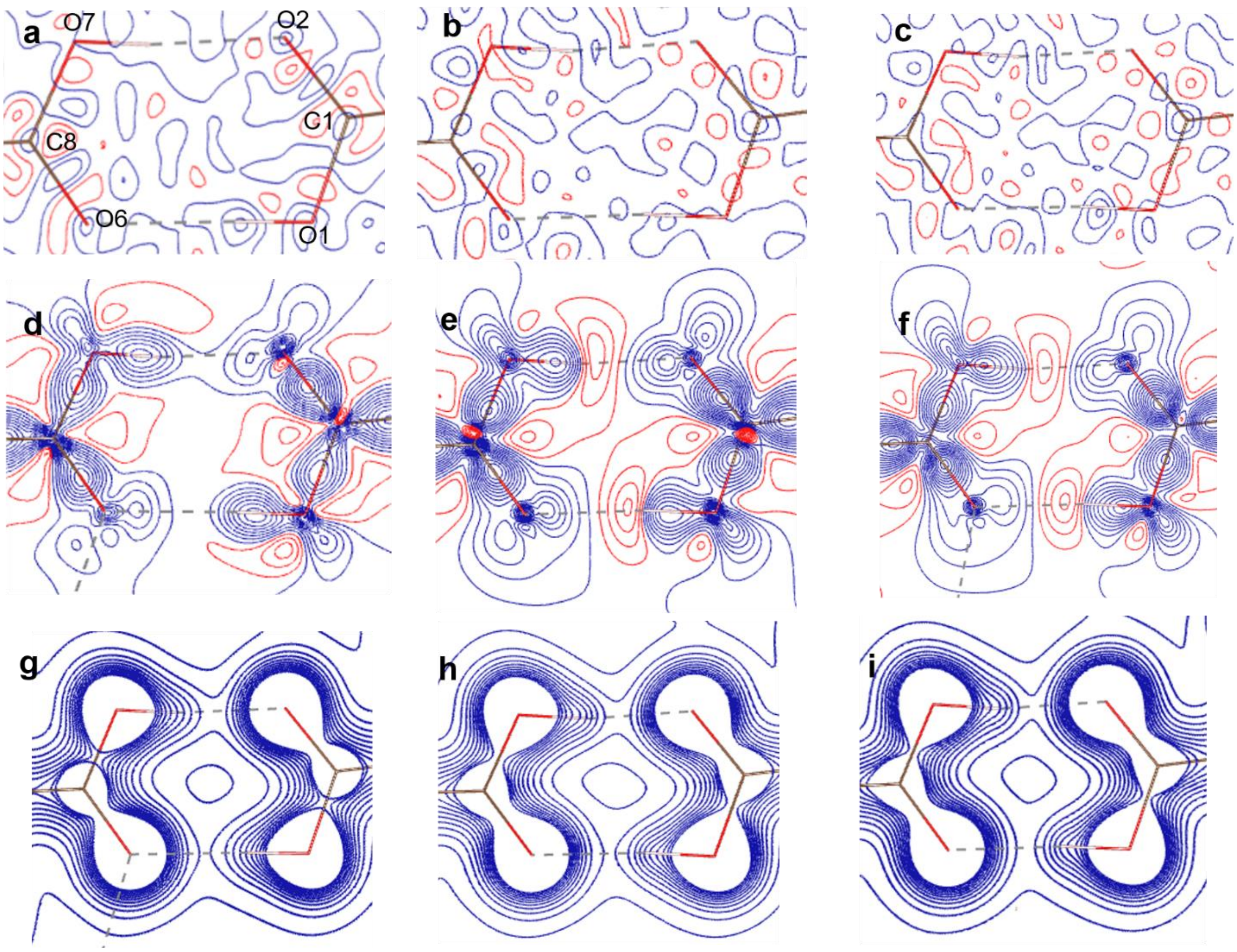

Figure S6. O1…O2…C8 plane of density maps of GPW cocrystal: 1st, 2nd and 3rd columns represent $\rho_{IAM}^{MEM-n4}(\boldsymbol{x})$, $\rho_{ELMAM2}^{MEM-n4}(\boldsymbol{x})$ and $\rho_{MP}^{MEM-n4}(\boldsymbol{x})$. (a), (b), (c) are residual density (difference Fourier maps) with contours at 0.1 eÅ^{-3}; (d), (e), (f) are dynamic deformation density with contours at 0.05 eÅ^{-3} and (g), (h), (i) are MEM density with contours at 0.1 eÅ^{-3}. Blue and red solid lines indicate positive and negative electron density, respectively.
